# Developmental trajectories of head and eye cue integration in gaze perception

**DOI:** 10.1038/s41598-025-34625-9

**Published:** 2026-01-06

**Authors:** Yumiko Otsuka, Katsumi Watanabe, Colin W. G. Clifford

**Affiliations:** 1 School of Psychology , Chukyo University , Nagoya, Japan; 2 Faculty of Law and Letters , Ehime University , Matsuyama, Japan; 3 School of Psychology , UNSW Sydney , Sydney, Australia; 4 Faculty of Science and Engineering , Waseda University , Tokyo, Japan

**Keywords:** Gaze perception, Face perception, Wollaston illusion, Head orientation, Adolescence, Cue integration, Neuroscience, Psychology, Psychology

## Abstract

Gaze perception is a foundational social skill. Here, we investigated how head and eye cue integration in gaze perception changes across development. Across five experiments involving 195 Japanese children (ages 4–16) and 126 adults (ages 18–58), we tested eye gaze perception using both Wollaston illusion images, where eye regions remain identical across head orientations, and Normal images with naturally varying eye regions. We found that the attractive influence of head orientation, whereby perceived gaze is biased toward the head direction, *decreased* from early childhood to adolescence. Notably, children aged 10–16 years did not show the attractive effect of head orientation characteristic of the Wollaston illusion. Adults consistently showed the illusion as expected. These findings highlight adolescence as a critical transitional period. A follow-up experiment with 7–9-year-old children showed that perceived gaze direction was more strongly biased toward head orientation in smaller images where iris and pupil positional details are less clearly visible, suggesting that the influence of head orientation is flexibly modulated by the clarity of eye region as well as age. The findings provide new insights into the dynamic development of social cue integration and perceptual decision-making across childhood, adolescence, and adulthood.

**Supplementary Information:**

The online version contains supplementary material available at 10.1038/s41598-025-34625-9.

## Introduction

Gaze perception is a foundational social skill. Perception of others’ gaze direction provides useful information such as the target of others’ attention and intentions during everyday interactions. The detectability of others’ gaze directions in human interaction is supported and enhanced by the unique external eye morphology of humans. The uniformly depigmented and largely exposed sclerae in human eyes ensure that the relative position of the pupil and iris within the eye opening is readily visible across various viewing conditions^1–4^. The simple luminance distribution pattern induced by the high contrast between the white sclera and the darker iris and pupil provides a robust cue to eye orientation^5^. Consequently, artificially altering the luminance distribution of the eye region in facial images, such as darkening the sclera on one side, can induce an illusory shift in perceived gaze direction toward the darkened side (the bloodshot illusion^6,7^).

**Fig. 1:**
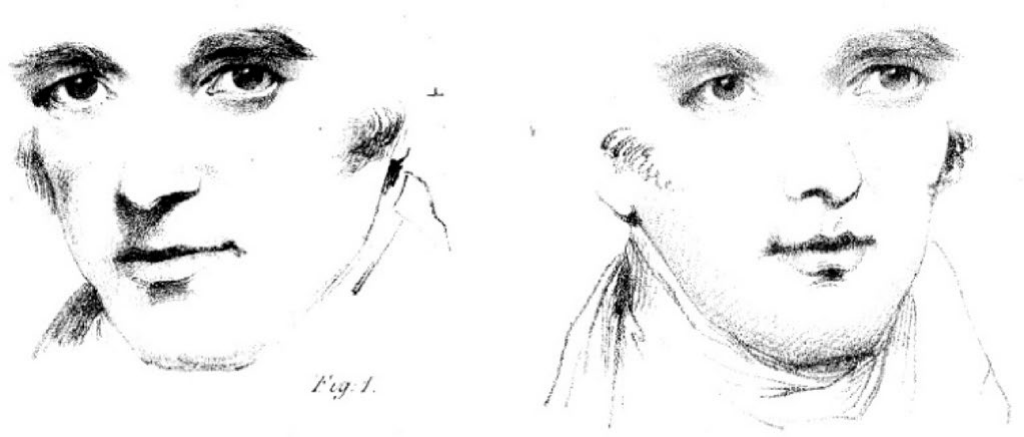
Demonstration by Wollaston^8^ (in the public domain).

While information available from the eyes is undoubtedly crucial for gaze perception, head orientation also plays a significant role. The importance of head orientation as a cue to gaze direction was first dramatically demonstrated by Wollaston^8^. Wollaston created a portrait by inserting identical eyes into drawings with different head orientations (Fig. 1). Although the two faces share identical eyes, they appear to be gazing in different directions, a phenomenon known as the Wollaston illusion.

**Fig. 2:**
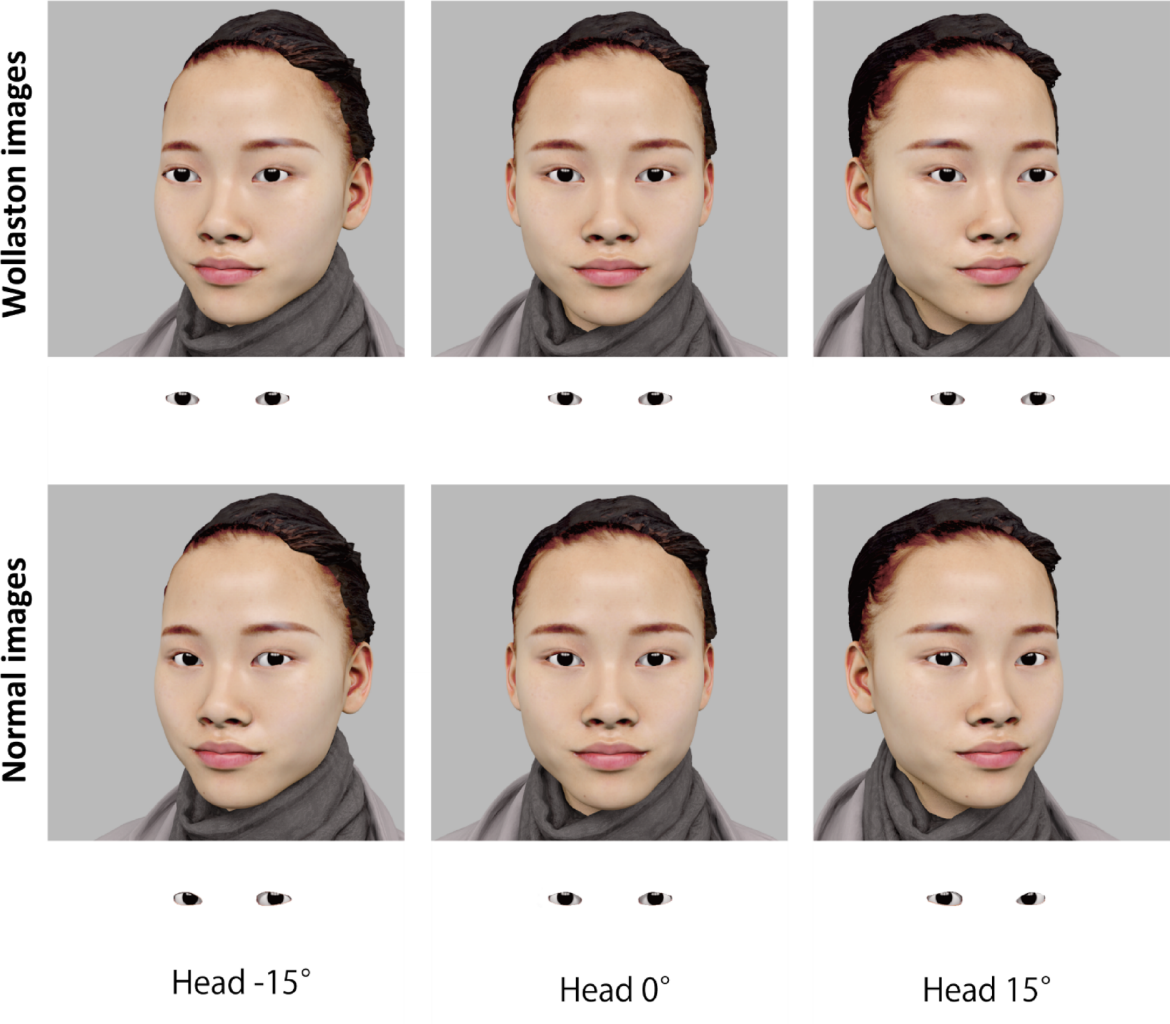
Examples of the Wollaston images and Normal images used in the current study. The eye regions below each image are for illustration only and were not used in the experiments. In the Wollaston images, the relative position of the pupil/iris within the eye opening remains constant across head orientations, whereas in the Normal images, the relative position of the pupil/iris shifts opposite to the head orientation to maintain the same eye orientation. The images shown here have a 0° eye orientation.

More recent studies have identified two ways in which the head’s orientation influences the perception of others’ eye gaze direction^9^. The first is the attractive influence observed in Wollaston images, where the perceived gaze direction shifts toward the same direction as the head orientation. Such an attractive effect is generally observed when stimulus eyes and head parts are two-dimensionally combined to produce facial images (see Fig. 2, top row^10–13^).

The second way in which head orientation influences gaze perception occurs when viewing faces in different head orientations. In such cases, the perceived gaze direction is biased in the direction opposite to the head orientation—a repulsive effect (e.g.^14–17^). This effect arises from changes in eye region information: when a head is rotated while the gaze remains fixed on a given point, the relative amount of visible sclera on either side of the iris shifts, mimicking a change in eye orientation opposite to the head rotation (Fig. 2, bottom row). This repulsive bias becomes most pronounced when only the eye region is visible during gaze judgment tasks^9,18^. When the whole face is visible, as in everyday face-to-face interactions, perceived gaze direction is influenced by two opposing effects of head orientation: a direct attractive influence (i.e., a Wollaston-illusion–like shift toward the head orientation) and a repulsive influence arising from changes in the eye region^9^. Although these two influences can partially cancel each other, studies with adults have consistently reported an overall repulsive effect of head orientation in such “Normal” face images (e.g.^9,14^).

Infant studies suggest that the integration of eye orientation and head orientation for gaze perception emerges during infancy. Newborns show a crude sensitivity to direct gazes, preferring them over an averted gaze or closed eyes^19,20^. However, this response is limited to frontally oriented faces and does not extend to angled faces at this stage^21^. By 4 months, infants can distinguish direct from averted gazes in angled faces^20,22^. Studies using Wollaston illusion images also suggested that 4- to 8-month-olds perceived different gaze directions from identical eyes placed in the varying head orientation context^23,24^.

After infancy, the ability to explicitly judge others’ gaze direction emerges around 3 years of age^25^ and is generally not observed in 2-year-olds. This lags somewhat behind the development of the ability to judge the direction of finger pointing and head orientation^25,26^. There is also evidence that young children make use of different types of information within the eye region^27^. Doherty et al.^27^ found that 3- and 4-year-olds used both geometric cues (i.e., circularity of iris and pupil) and luminance cues when explicitly judging gaze direction, performing better with full-band grayscale faces than with blurred or line-drawn faces. They also showed that gaze-following in 2- and 3-year-olds relied strongly on luminance-based information, with cueing effects observed for full-band and blurred faces but not for line-drawn faces.

Several studies using psychophysical tasks with children older than 4 years old have shown that fine-grained sensitivity to eye gaze continues to develop throughout childhood. For example, Vida and Maurer^28^ reported that the gaze discrimination thresholds in 6- and 8-year-olds were approximately twice those of adults, but the thresholds were comparable to adult levels in 10- and 14- years of age. Other studies have reported that compared to children older than 10 years and adults, younger children judge a wider range of eye orientations as gazing directly toward them^29,30^.

Moreover, it has been suggested that younger children rely more on head orientation when judging gaze directions^31,32^, which is of particular relevance to the present study. Mihalache et al.^31^examined the relative weighting of eye and head orientations in gaze perception among typically developing children and those with autism spectrum disorder (ASD). They observed that across 7- to 15-year-olds, the reliance on head orientation decreased with age, regardless of ASD status. Although this finding by is insightful, several limitations should be noted. First, it may partly reflect the small stimulus size (~ 2.3° visual angle). Although visual acuity nears adult levels by age 6, younger children experience greater foveal crowding (e.g.^33^). This would make it harder for younger children to detect small pupil shifts. In addition, confusion about the task may have affected children’s responses. Specifically, interleaved head orientations may have led to confusion in the young children about whether to judge eye gaze direction or head orientation.

To examine developmental changes in the integration of head and eye orientation cues in gaze perception, we tested 4- to 16-year-olds using Wollaston images (Experiment 1) and normal images (Experiment 3) (Fig. 2). To ensure visibility of the eyes for younger children, we used large facial images, and provided practice trials to confirm task understanding. Further, we presented the three head orientations in separate blocks for ease of comprehension, and head orientation was fully manipulated within subjects. Adults were tested under the same conditions (Experiments 2 and 4) for comparison. Finally, we assessed whether the size of the facial image affects how children integrate head and eye orientation cues in gaze perception (Experiment 5).

## Results

### Experiment 1: children’s perception of Wollaston images

In Experiment 1, we tested how head orientation affects children’s gaze perception by using Wollaston images (Fig. 2, top row), where the eye region remains unchanged across three head orientations. If the children’s judgments rely only on eye region information, the head orientation should have no effect. However, if the Wollaston illusion occurs, the perceived gaze should shift toward the head orientation, indicating that head orientation acts as a direct cue for gaze perception^9^.

**Fig. 3:**
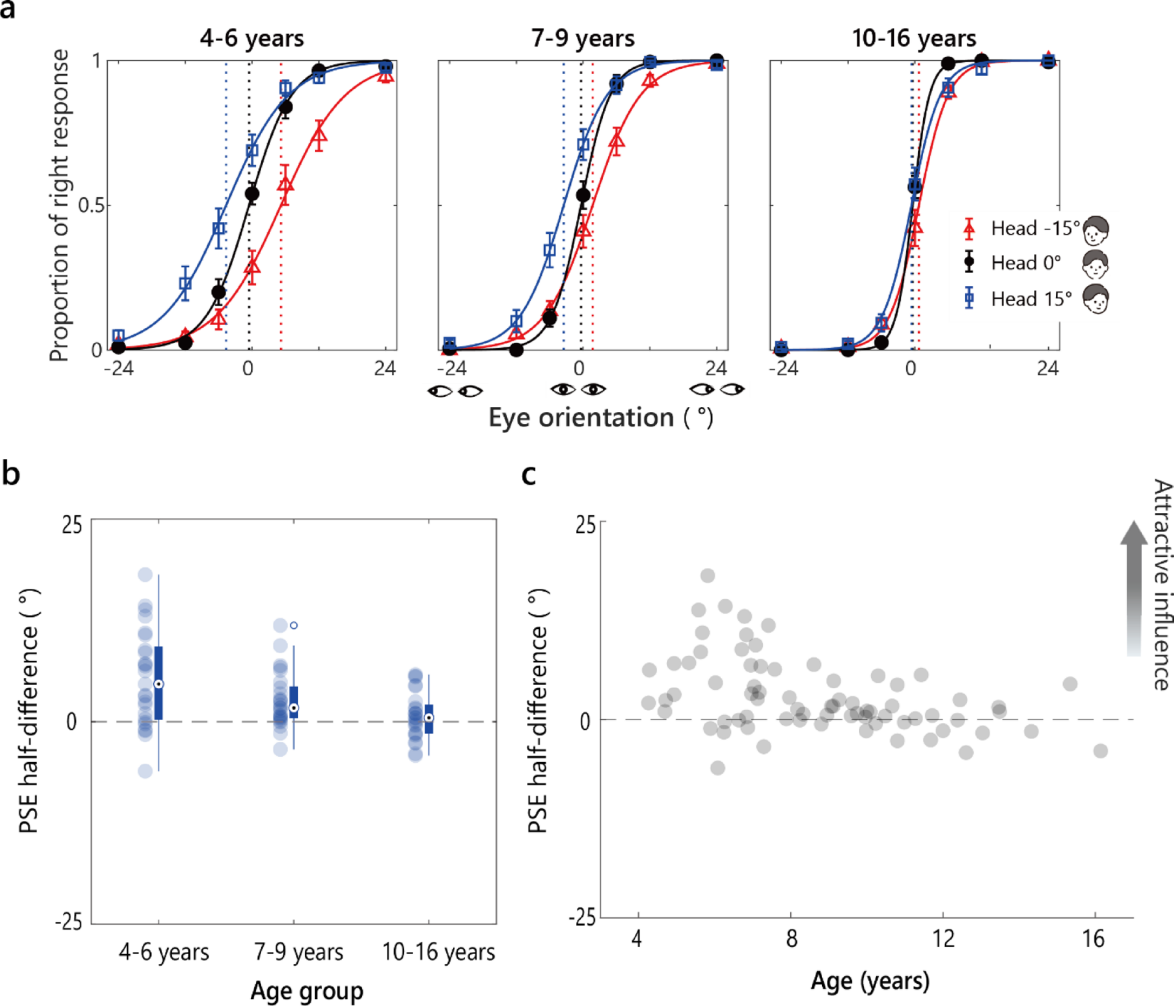
Results from Experiment 1 (Wollaston images). **a** The average proportion of rightward responses per age group is plotted against the stimulus eye orientation for each head orientation. Solid curves show logistic fits. Vertical dashed lines mark the estimated PSEs. Error bars represent ± 1 SEM (standard error of the mean). **b** The PSE half-difference for each age group. Filled circles represent individual data points. Box plots show the median and interquartile range (IQR). Whiskers extend to values within 1.5 × IQR, and open circles denote outliers. **c** The individual PSE half-difference values plotted as a function of age collapsed across age groups.

For each eye orientation and face orientation, we calculated each participant’s proportion of rightward responses. A logistic function was fitted to these proportions by eye orientation for each head orientation. Figure 3a shows the average rightward response proportions per age group with logistic fits. Vertical dotted lines indicate the point of subjective equality (PSE: 50% rightward responses) for each head orientation. We then calculated each participant’s PSE half-difference, calculated as the average shift from 0 of the PSEs for the leftward and rightward head orientations.

Figure 3b shows the PSE-half difference for each age group. Positive values indicate an attractive shift of the perceived gaze toward the head orientation, and negative values indicate a repulsive shift away from the head orientation. The PSE half-difference tended to be positive in the younger groups but was near zero in the 10- to 16-year-olds. A Welch analysis of variance (ANOVA) revealed a significant main effect of age, *F* (2, 45) = 6.51, *p* =.003, *η*²_p_ =.16. A post hoc analysis with a Holm-Bonferroni correction showed that the PSE half-difference was significantly more positive in the 4- to 6-year-olds than in the 10- to 16-year-olds (*p* =.001, *d =* 1.06). No significant differences were observed between other age groups (4–6 years vs. 7–9 years, *p* =.068, *d =* 0.61; 7–9 years vs. 10–16 years, *p* =.125, *d =* 0.44), suggesting a stronger attractive influence in the younger children.

To test for the Wollaston illusion, we used one-sample *t*-tests to compare the PSE half-differences against 0 (chance level). The PSE half-difference was significantly greater than 0 in the two younger age groups: 4–6 years: *M* = 5.29, *SD* = 5.99, *t* (24) = 4.42, *p =.*001, *d =* 0.88; 7–9 years: *M* = 2.63, *SD* = 3.48; *t* (24) = 3.78, *p =*.002, *d =* 0.76. These results confirm that younger children (4–9 years) perceived the Wollaston illusion. By contrast, in the 10- to 16-year-olds, the PSE half-difference was unaffected by head orientation (*M* = 0.70, *SD* = 2.90, *t* (23) = 1.19, *p* =.25, *d =* 0.24), providing no evidence of the Wollaston illusion in this age group.

We also performed a Spearman’s rank-order correlation to assess the relationship between the individual PSE half-difference values and age (Fig. 3c). The analysis revealed a significant negative correlation ( = −0.409, *p* <.001), confirming that the PSE half-difference decreased with age, which was consistent with the group-level trends.

### Experiment 2: adults’ perception of Wollaston images

The results of Experiment 1 demonstrated that the Wollaston illusion weakened across childhood and was minimal by adolescence. Given previous studies showing a robust Wollaston illusion in adults^10–13,18^, our results suggest that the developmental trajectory of head and eye cue integration is not strictly monotonic. Rather than progressively approaching the adult pattern, adolescents showed the weakest illusion in our sample, indicating a possible transitional reweighting of head and eye cues during this period. To confirm that adults perceive the Wollaston illusion as reported by previous studies and to explore potential developmental changes in the perception of the illusion in adulthood, we conducted Experiment 2 using Wollaston images to test adults. The participants were divided into three age groups: undergraduate students around 20 years of age, adults in their 20 s and 30 s, and adults in their 40 s and 50s.

**Fig. 4:**
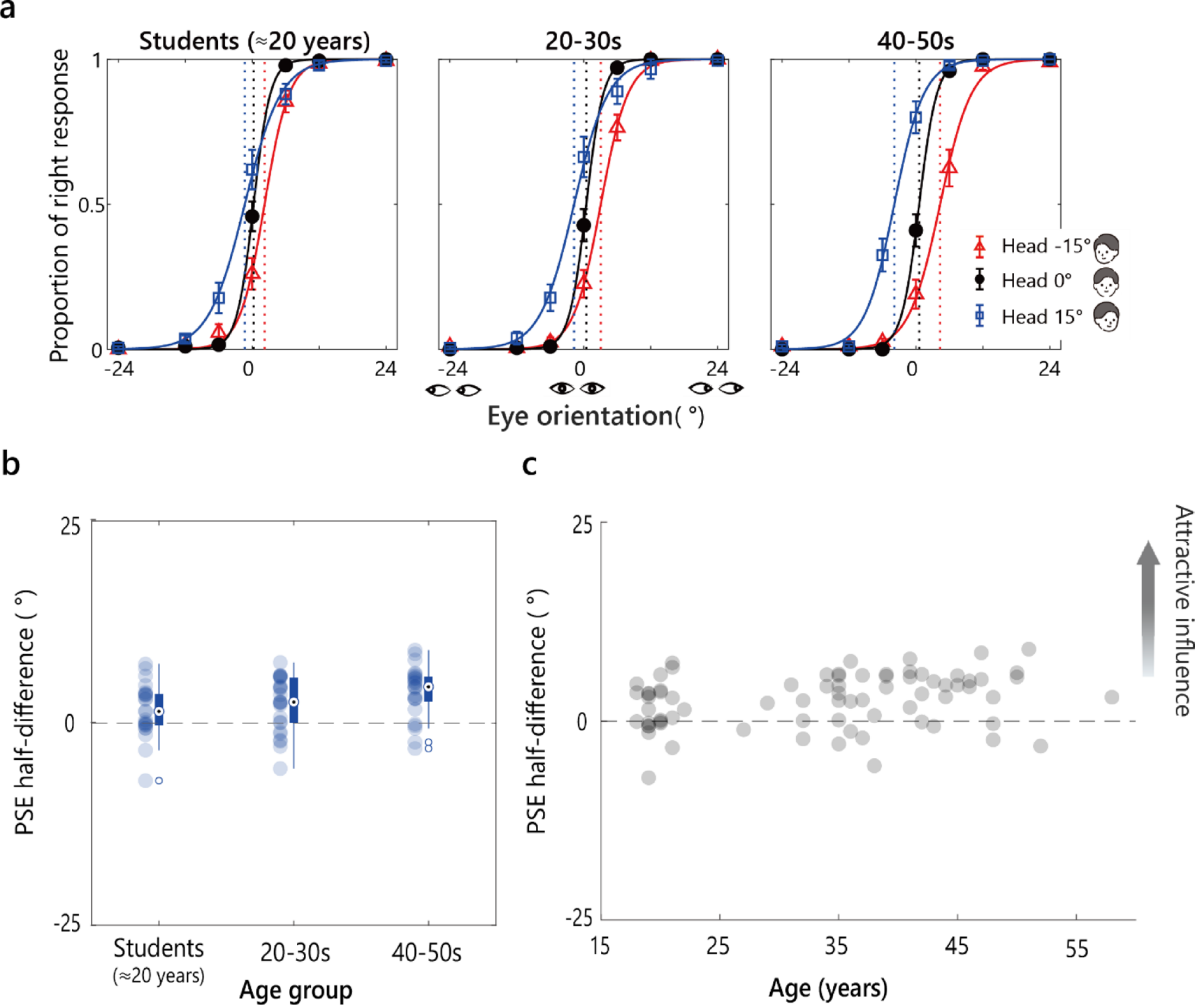
Results from Experiment 2 (Wollaston images). **a** The average proportion of rightward responses per age group is plotted against the stimulus eye orientation for each head orientation. Solid curves show logistic fits. Vertical dashed lines mark the estimated PSEs. Error bars represent ± 1 SEM. **b** The PSE half-difference for each age group. Filled circles represent individual data points. Box plots show the median and IQR. Whiskers extend to values within 1.5 × IQR, and open circles denote outliers. **c** The individual PSE half-difference values plotted as a function of age collapsed across age groups.

Figure 4a shows the logistic fits to the average proportion of rightward responses in each age group. As observed for the younger children in Experiment 1, the adult response curves shifted opposite to the head orientation.

Figure 4b presents the PSE half-difference by age group. A Welch ANOVA did not show a main effect of age on the PSE half-difference: *F* (2,47) = 3.12, *p* =.053, *η*²_p_ =.078. One-sample *t*-tests confirmed that the PSE half-difference was significantly greater than 0 in all three age groups: students **≈** 20 years, *M =* 1.59, *SD* = 3.27, *t* (23) = 2.39, *p* =.026, *d =* 0.49; 20–39 years, *M =* 2.37, *SD* = 3.38, *t* (25) = 3.57, *p* =.003, *d =* 0.71; and 40–59 years, *M =* 3.86, *SD* = 3.18, *t* (24) = 6.08, *p* <.001, *d =* 1.22. These results suggest that the perceived gaze direction was consistently attracted toward the head orientation across all adult age groups, confirming the perception of the Wollaston illusion in adulthood with the stimuli and experimental set used here.

As in Experiment 1, we performed a Spearman’s correlation between the PSE half-difference values and age (Fig. 4c). it revealed a significant positive correlation ( = 0.272, *p* =.018). This suggests an age related trend that was undetectable at the group level, where the PSE half-difference increased with age. That is, the effect of head orientation increased with age in adulthood.

### Experiment 3: children’s perception of normal images

In Experiment 3, we examined the effect of head orientation on eye gaze perception in children using Normal images (Fig. 2, bottom row). In these images, perceived gaze direction emerges as the combination of two opposing influences of head orientation: an attractive influence as seen in the Wollaston illusion, and a repulsive influence arising from the changes in eye region information. Because these influences can partly cancel each other out, the magnitude of the overall head orientation effect depends on the relative weighting of head and eye cues. In Experiment 1, we found that the attractive influence of head orientation decreased with age during childhood. We therefore reasoned that, if the repulsive influence remained relatively stable across age, the net effect of head orientation would be increasingly more repulsive in older children.

**Fig. 5:**
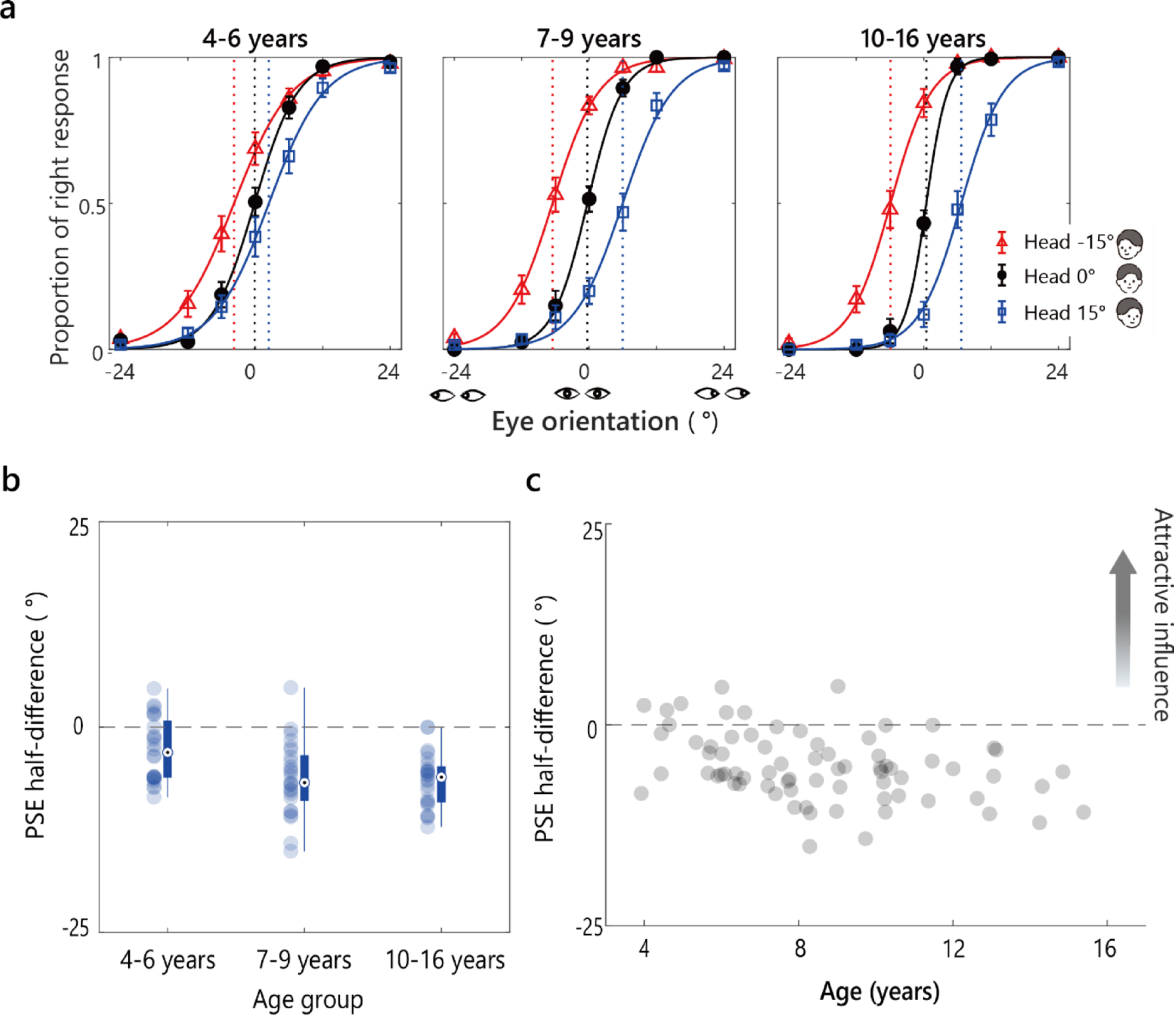
Results of Experiment 3 (Normal images). **a** The average proportion of rightward responses per age group is plotted against the stimulus eye orientation for each head orientation. Solid curves show logistic fits. Vertical dashed lines mark the estimated PSEs. Error bars represent ± 1 SEM. **b** The PSE half-difference for each age group. Filled circles represent individual data points. Box plots show the median and IQR. Whiskers extend to values within 1.5 × IQR, and open circles denote outliers. **c** The individual PSE half-difference values plotted as a function of age collapsed across age groups.

Figure 5a shows the logistic fits to the average proportion of rightward responses in each age group. The PSE half-difference was generally negative, with a stronger repulsive effect in older children (Fig. 5b). A Welch ANOVA revealed a significant main effect of age: *F* (2, 46) = 7.09, *p* =.002, *η*²_p_ =.16. Post hoc tests with Holm–Bonferroni correction showed that the PSE half-difference was significantly more positive in the 4- to 6-year-olds than in older children (vs. 7–9 years: *p* =.006, d = 0.879; vs. 10–16 years: *p* =.005, *d =* 0.947). No significant difference was observed between the two older groups (*p* =.81, *d =* 0.07). One-sample *t*-tests on the PSE half-difference against 0 for each age group showed that the PSE half-difference was significantly below 0 in all age groups (4–6 years: *M* = − 2.82, *SD* = 3.87, *t* (23) = − 3.58, *p* =.002, *d = −* 0.73; 7–9 years: *M* = − 6.26, *SD* = 4.48, *t* (24) = − 6.99, *p* <.001, *d = −* 1.40; 10–16 years: *M* = − 6.53, *SD* = 3.27, *t* (23) = − 9.77, *p* <.001, *d = −* 2.00).

These results indicate that in Normal images, where changes in head orientation induce natural repulsive shifts in the relative position of the iris/pupil, the perceived gaze direction was repelled opposite to the head orientation. The less negative PSE half-difference in the 4- to 6-year-olds aligns with the results of Experiment 1, in which a stronger attractive influence of head orientation was observed in younger children. This suggests that the repulsive effect that occurred in Experiment 3 was mitigated by a stronger attractive influence in younger children.

As in Experiment 1, Spearman’s rank-order correlation assessing the relationship between individual participants’ PSE half-difference values and their ages revealed a significant negative correlation ( = −0.346, *p* =.003; Fig. 5c). These results suggest that the individual PSE half-difference tended to decrease with age, confirming the trend observed in the group-level analysis.

### Experiment 4: adults’ perception of normal images

In Experiment 4, we used Normal images (Fig. 2, bottom row) to examine the influence of head orientation on eye gaze perception in adults. Since no significant differences were observed between the adult age groups in Experiment 2, we tested only two groups: students aged ≈ 20 years and adults aged 20–59 years.

**Fig. 6:**
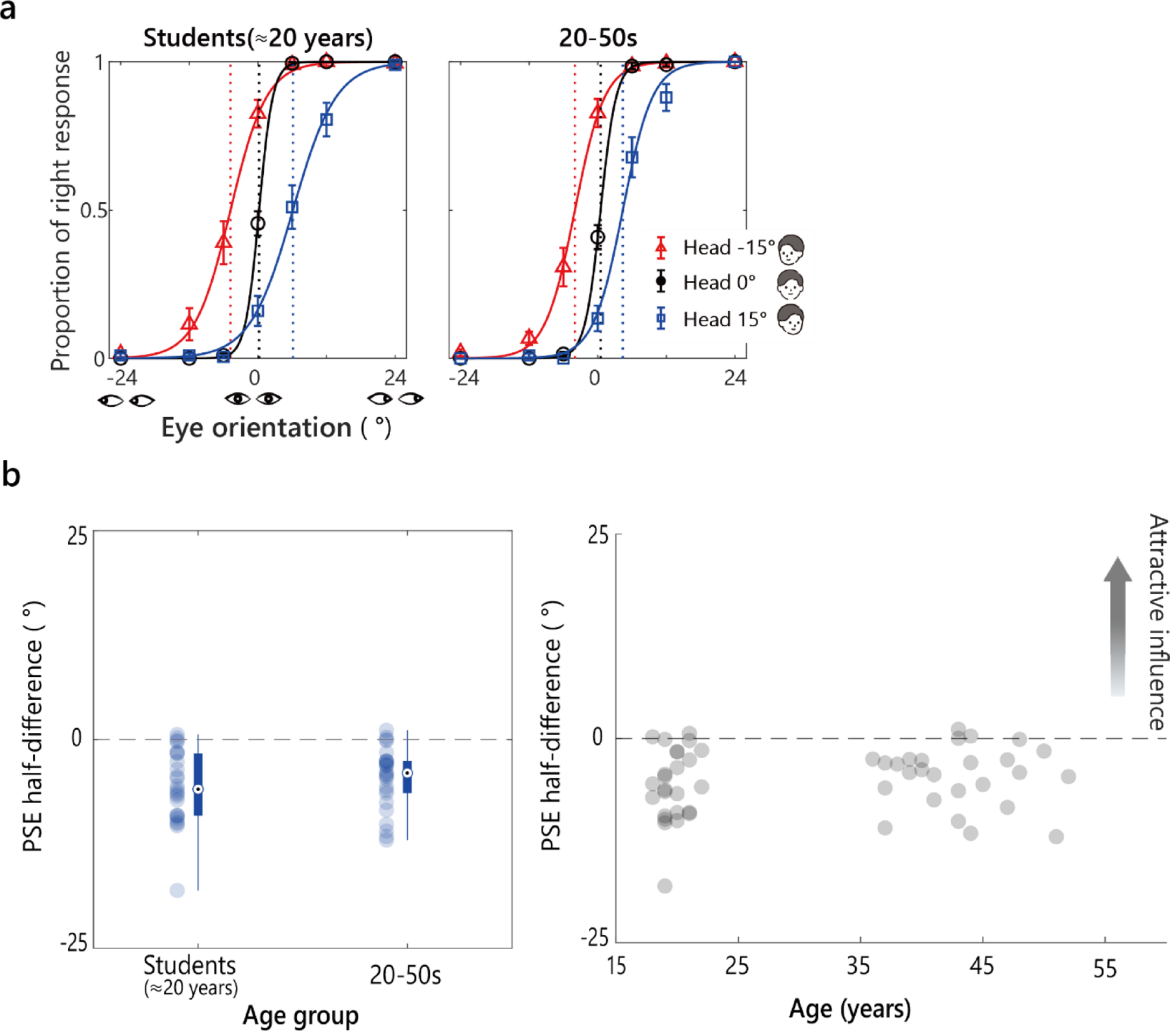
Results of Experiment 4 (Normal images). **a** The average proportion of rightward responses per age group is plotted against the stimulus eye orientation for each head orientation. Solid curves show logistic fits. Vertical dashed lines mark the estimated PSEs. Error bars represent ± 1 SEM. **b** The PSE half-difference for each age group. Filled circles represent individual data points. Box plots show the median and IQR. Whiskers extend to values within 1.5 × IQR, and open circles denote outliers. **c** The individual PSE half-difference values plotted as a function of age collapsed across age groups.

Figure 6a shows the logistic fits to the average proportion of rightward responses. The PSE half-difference was generally negative in both age groups (Fig. 6b). An independent *t*-test comparing the two groups showed no significant difference: *t* (49) = 0.97, *p* =.335, *d =* 0.273. One-sample *t*-tests against 0 revealed that the PSE half-difference was significantly negative in both groups (students ≈ 20 years: *M* = − 5.72, *SD* = 4.42, *t* (24) = − 6.48, *p* <.001, *d = −* 1.30; 20–59 years: *M* = − 4.62, *SD* = 3.67, *t* (25) = − 6.42, *p* <.001, *d = −* 1.26). As in an earlier investigation (e.g., Otsuka et al. 2014, 2015), these results demonstrate a repulsive influence of head orientation on perceived gaze direction in the Normal images.

A Spearman’s rank-order correlation assessing the relationship between the individual participants’ PSE half-difference values and their ages revealed no significant correlation ( = 0.154, *p* =.281; Fig. 6c). This may be attributable to the smaller number of participants than in Experiment 2.

### Experiment 5: children’s perception of small Wollaston and normal images

In Experiment 5, we explored the possibility that the way children rely on these cues is flexibly modulated depending on the stimulus condition. A previous psychophysical study with adults reported that adults relied more on head orientation under peripheral viewing conditions, where detailed eye information is harder to extract^34^. Based on such findings, we explored whether children’s reliance on head and eye cues varies with the clarity of the eye region cue. We tested this by presenting smaller Wollaston and Normal images to children, which made it more difficult to discern iris and pupil positional details. Due to the higher number of 7- to 9-year-old participants enrolled during the participant recruitment, we focused on this age group in Experiment 5.

**Fig. 7:**
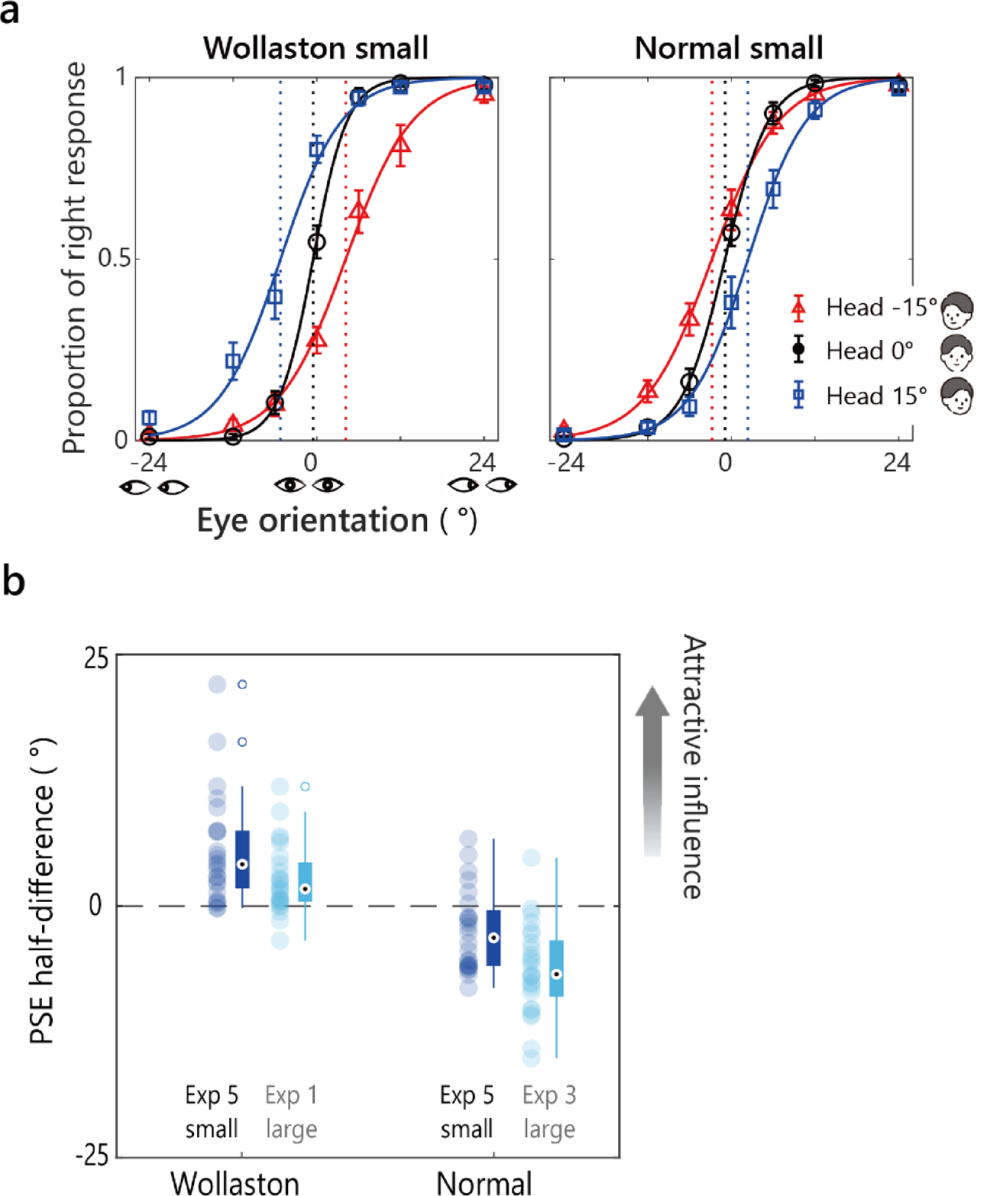
Results of Experiment 5 (small images). **a** The average proportion of rightward responses per image condition is plotted against the stimulus eye orientation for each head orientation. Solid curves show logistic fits. Vertical dashed lines mark the estimated PSEs. Error bars represent ± 1 SEM. **b** The PSE half-difference for each condition, shown together with data from the same age group in Experiment 1 (Wollaston images) and Experiment 3 (Normal images) using large images. Filled circles represent individual data points. Box plots show the median and interquartile range (IQR). Whiskers extend to values within 1.5 × IQR, and open circles denote outliers.

Figure 7a shows the logistic fits to the averaged proportion of rightward responses, and Fig. 7b presents the PSE half-difference values for each image condition. One-sample t-tests revealed significant shifts: positive in the Wollaston condition (*M =* 5.54, *SD* = 5.48, *t* (23) = 4.95, *p* <.001, *d =* 1.01) and negative in the Normal condition (*M = −* 2.53, *SD* = 4.07, *t* (23) = − 3.04, *p* =.006, *d = −* 0.621). These results align with those of the previous experiments in this study, indicating an attractive influence of head orientation in the Wollaston condition and a repulsive influence in the Normal condition.

To examine the impact of the image size on the influence of head orientation, we compared Experiment 5 (small images) with the 7- to 9-year-olds group in Experiment 1 (large Wollaston images) and Experiment 3 (large Normal images). A two-way ANOVA with image size (Large vs. Small) and image condition (Wollaston vs. Normal) as between-subject factors revealed significant main effects of the image condition, i.e., *F* (1, 94) = 89.81, *p* <.001, *η*²_p_ =.489 and the image size, i.e., *F* (1, 94) = 13.78, *p* <.001, *η*²_p_ =.128, but no significant interaction: *F* (1, 94) = 0.22, *p* =.642, *η*²_p_ =.002. A stronger attraction to head orientation in the Wollaston images compared to the Normal images explains the main effect of the image condition. The more positive PSE half-difference for smaller images suggests a stronger attractive effect of head orientation when eye region details were less visible. These findings indicate that children flexibly adjust their reliance on head and eye cues based on the cues’ visual availability.

## Discussion

We examined the integration of head and eye orientation cues in gaze perception for children (ages 4–16 years) and adults (aged 18–59 years). We consistently observed that the attractive influence of head orientation diminished with the participants’ age during childhood to adolescence. This was evident in both Wollaston images (Experiment 1) and Normal images (Experiment 3). Notably, the Wollaston illusion was absent in the 10- to 16-year-olds. No significant age differences were observed among the adults, although the illusion was somewhat weaker in the youngest adults (~ 20 years old). The PSE shift tended to be smaller in this group, with the smallest effect size (*d* = 0.49; ages 20–39 years: *d* = 0.70; ages 40–59 years: *d* = 1.22). An additional reaction-time analysis (Supplementary Note 1) also showed that neither the adolescents nor the young adults exhibited systematic peak shifts across head orientations, unlike younger children and older adults. These findings indicate a U-shaped developmental trajectory in which the influence of head orientation is least pronounced during adolescence. Experiment 5 further demonstrated that the weighting of head information in gaze perception can be modulated by stimulus properties even within a single age group.

To ensure task comprehension, we provided practice trials, used objective rejection criteria, and presented each head orientation in a separate block to reduce task confusion and maintain the focus on judging eye gaze. We also used large facial images in order to maximize eye visibility even for young children. We observed the developmental changes in the use of head and eye orientation cues, despite these controls. These controls rule out the possibility that the observed developmental differences can be attributed to variations in task comprehension, task engagement, or stimulus visibility across age groups.

Although our task required left–right gaze discrimination rather than explicit identification of direct gaze, reaction-time analyses (Supplementary Note 1) revealed delayed responses for perceptually direct gaze. Consistent with prior findings^13,35^, direct gaze elicited slower reaction times, likely due to increased decision difficulty. Notably, we observed this delay most prominently for the frontally oriented faces, suggesting that direct gaze perception is particularly salient when both the head and the eyes align toward the observer. Moreover, the participants’ reaction time patterns mirrored our PSE findings, reinforcing the observed developmental trends. The shifts in peak reaction times across head orientations suggested an attractive influence of head orientation in Wollaston images and a repulsive influence in Normal images. This effect was less pronounced in the 10- to 16-year-olds and in the ≈ 20-year-old student group, suggesting reduced head orientation weighting in these groups.

We observed that the influence of head orientation declined systematically from early childhood to adolescence. This is consistent with previous reports that younger children relied more on head orientation cues in gaze judgement. For example, Doherty et al.^25^ found that 3- to 6-year-olds judged gaze directions more accurately when the experimenter’s head was aligned with the target rather than when only the eyes shifted. Mihalache et al.^31^ similarly reported a reduction in head orientation influence between 7 and 15 years of age. Because their study used small facial stimuli (~ 2.3° visual angle), younger children may have had reduced ability to detect fine-grained eye region cues, which could have led to an increase in their relative reliance on head orientation. In contrast, the present study used large, highly visible images that ensured clear perception of both eye and head cues even in the youngest children. Despite this improved visibility, we still observed a systematic decline in head weighting with age, reaching its minimum during adolescence. This suggests that the developmental change reflects a genuine reorganization in how head and eye cues are integrated, rather than differences in stimulus visibility.

Our results suggest that eye region information becomes relatively more salient around adolescence. Despite the identical stimuli and test conditions, the attractive influence of head orientation was weakest in the 10- to 16-year-olds. In fact, they were able to judge the gaze direction based solely on eye region cues, without susceptibility to the Wollaston illusion (Experiment 1).

Why is the influence of eye region cues more pronounced in adolescence? One possibility is that adolescents have higher precision in processing eye region information. In cue combination, perceptual systems integrate multiple cues by weighting each cue according to its relative reliability; a more reliable cue—one that supports more precise discrimination—receives greater weight in the final perceptual estimate. If eye region cues become especially reliable during adolescence, then reduced head weighting would follow. To examine this possibility, we compared the slope of the psychometric functions for the gaze discrimination task across age groups (Supplementary Note 2). We found that, for both Wollaston and Normal images, slopes were significantly shallower in the 4- to 6-year-olds and 7- to 9-year-olds compared with adults, whereas the 10- to 16-year-olds did not differ significantly from adults. These findings suggest better precision of gaze discrimination in 10- to 16-year-olds and adults than in younger children. This aligns with Vida and Maurer^28^, who reported that gaze discrimination thresholds in 10- and 14-year-olds are comparable to adults, whereas thresholds are higher in 6- and 8-year-olds. Given that we did not find a difference in perceptual precision in eye gaze processing between adolescents and adults, the precision alone cannot account for the reduced influence of head orientation in adolescence.

Adolescence is widely viewed as a transitional period marked by substantial changes in social motivation, affective processing, and neural systems supporting social cognition^36^. During this period, sensitivity to social evaluation and others’ attention increases markedly. For example, the mere presence of peers, or the belief that someone is watching them exerts a stronger influence on adolescents’ behavior and self-awareness than on adults or younger children^37–40^. At the neural level, regions involved in social emotional processing show pronounced developmental changes, with the limbic system including the amygdala exhibiting heightened responsivity to socially salient cues (e.g.^41–46^). The amygdala plays a key role in guiding attention toward the eye region of faces^47–49^, even though it may not directly encode gaze direction^50^. This developmental profile suggests that adolescents may be especially attuned to information in the eyes. We therefore speculate that the reduced weighting of head orientation cues observed in adolescence, relative to both younger children and adults, may reflect the heightened salience of the eyes during a period in which social emotional processing is undergoing rapid reorganization.

We also found that the influence of head orientation on gaze perception was modulated by stimulus size. Smaller facial images increased reliance on head cues among 7- to 9-year-olds (Experiment 5). The large face stimuli (9.45°) used in Experiments 1–4 correspond to viewing a real face from approximately 85 cm. This falls within Hall’s^51^ personal interaction zone and within empirically preferred interpersonal distances for children and adults^52^. In contrast, the small face stimuli (2.36°) correspond to viewing a face from about 3.4 m. This distance lies near the boundary between Hall’s social and public distance zones, characteristic of classrooms, playgrounds, or other group settings in which gaze is monitored from afar. Thus, our large and small image conditions represent distinct but ecologically meaningful viewing contexts. Both sizes fall within the stimulus size range used in prior gaze perception studies, which have employed facial stimuli approximately 7–15° (e.g.^14,18,24,28–30,34^) and, in other cases, much smaller images of 1.6–4° (e.g.^15,16,25,31^). Our findings demonstrate that even within this ecologically valid range, cue weighting remains flexible, underscoring the adaptive nature of gaze-cue integration.

A limitation of the present study is that the findings were obtained within a single cultural context. All participants in the current study were Japanese. In addition, data collection took place during a period when mask wearing was nearly ubiquitous in public due to the COVID-19 pandemic. This social situation at the time of data collection may also have altered everyday exposure to facial cues. Nevertheless, the overall pattern of results observed in adults—namely, the presence of a Wollaston illusion and a repulsive effect in Normal images—is consistent with previous studies conducted in other countries prior to the COVID-19 (e.g.^9,18^). Likewise, the developmental pattern identified here—a reduction in the influence of head orientation from childhood to adolescence—is broadly in line with pre-pandemic findings from different cultural contexts (e.g.^31^). Even so, future cross-cultural research will determine the extent to which these developmental patterns generalize across cultures and social environments.

In summary, the present study reveals a U-shaped developmental trajectory in the integration of head and eye cues in gaze perception, highlighting adolescence as a critical transitional period. Moreover, our results show that even in children, the reliance on head and eye cues was flexibly modulated depending on the clarity of the cues, suggesting the dynamic nature of gaze cue integration both across development and within individuals at each age.

## Methods

### Ethics statement

All experiments were approved by the Ethics Committee for Human Research, Faculty of Law and Letters, Ehime University (approval no. 2021-15), and conducted in accordance with the Declaration of Helsinki. The data reported herein were collected from October 2021 to February 2023 at Ehime University and at *Himitsu ja Nai Kichi* (translated as “Not a Secret Base”), a cultural and educational community facility associated with the Dogo Onsen Art project in Matsuyama, Japan. We obtained written informed consent from all adult participants and legal guardians of the participating children.

### Apparatus

**Fig. 8:**
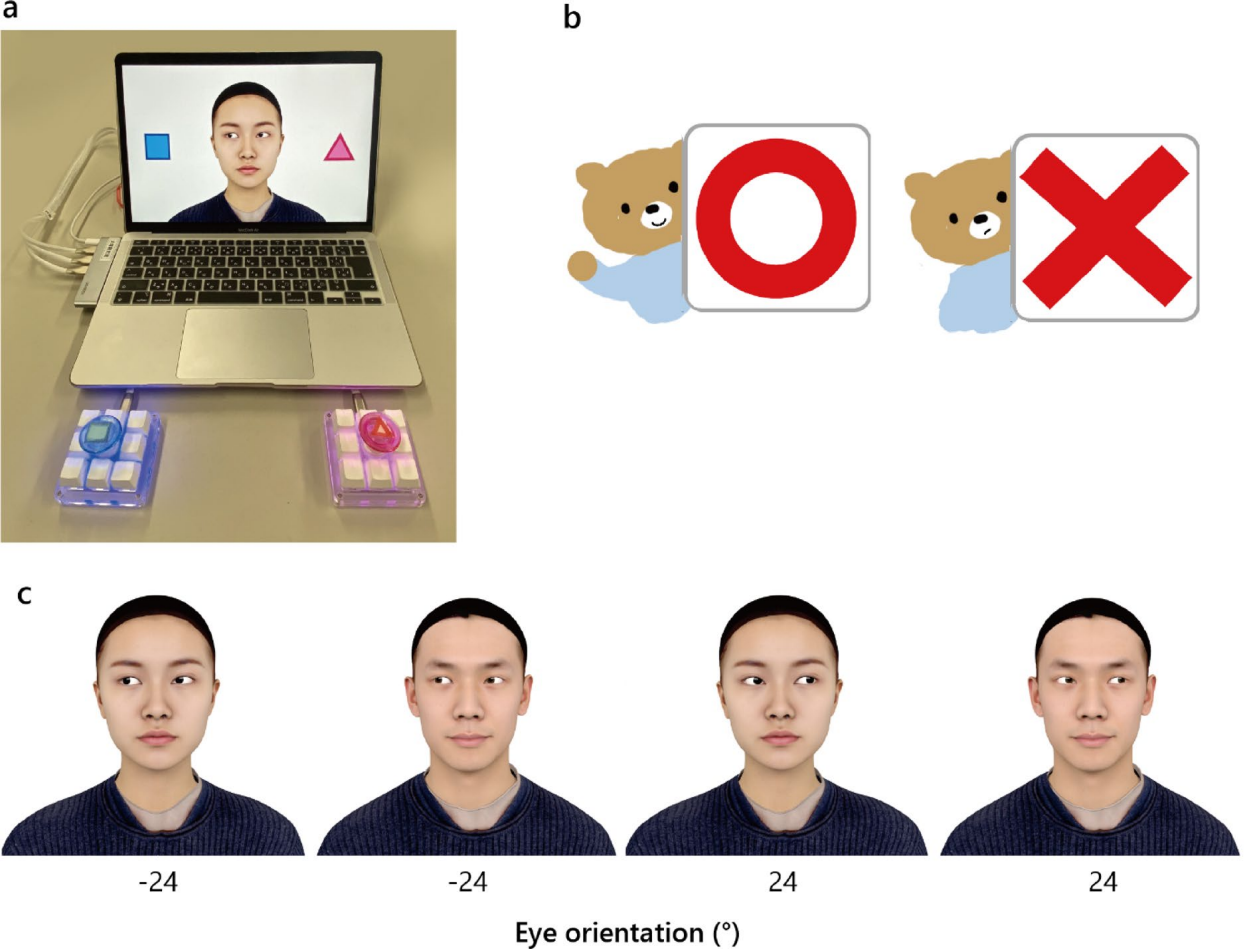
Equipment and stimuli for the practice trials. **a** Equipment used in the current study, displaying a practice trial stimulus on the screen. **b** Illustration of the correct (left) and incorrect (right) feedback images used in the practice trials, accompanied by the short “Pin-Pon” and “Boo” sounds during testing. **c** Stimulus face images used in the practice trials.

Stimuli were presented on the screen of a MacBook Air and controlled by Psychopy^53^. Two external mechanical keyboards were used to collect the participants’ responses (Fig. 8a). Although each keyboard had nine keys, all but the central key were disabled. Each keyboard was set to emit either a blue or pink light, and a large button cap of the corresponding color was placed on the central key. A blue-square sticker and a pink-triangle sticker were attached to the blue and pink button caps, respectively.

### Preregistration

This study was not preregistered.

### Use of generative AI tools

During the preparation of this manuscript, the authors used ChatGPT^54^ to improve the clarity and readability of the English expressions in this manuscript.

### Experiment 1

#### Participants

Twenty-five 4- to 6-year-olds (*M* = 5.88 years, *SD* = 0.86; 13 females), 25 7- to 9-year-olds (*M* = 8.41 years, *SD* = 1.00; 12 females), and 24 10- to 16-year-olds (*M* = 12.20 years, *SD* = 1.97; 12 females) participated in this experiment. An additional 24 children were excluded due to incomplete tasks (*n* = 9), failing to reach 75% accuracy for the most averted gaze in any testing block (*n* = 14), or equipment failure (*n* = 1).

We grouped participants aged 10–16 years together based on prior findings showing that gaze-perception performance becomes relatively stable from around 10 years of age. Mihalache et al. (2018) reported a gradual increase in the weighting of eye orientation across 7–15-year-olds without subdividing the sample into age groups, and Vida and Maurer (2012) found that 10- and 14-year-olds demonstrated adult-like gaze discrimination thresholds, unlike younger children aged 6 and 8 years. Additionally, we tested younger children from age 4 onward, as psychophysical gaze discrimination tasks become readily applicable at this developmental stage.

#### Stimuli

Using 3D head models of four individuals, we rendered face images with 7 eye orientations (− 24°, − 12°, − 6°, 0°, 6°, 12°, and 24°) with 3 head orientation pose (−15°, 0°, 15°). To create Wollaston images (Fig. 2, top row), we extracted the eye region from a frontal face and overlaid it onto the same face with different head orientations, ensuring identical eye regions across three orientations for each eye orientation. These images, presented on a light gray background, were used in the main task trials. Faces in the frontal orientation were 9.45 cm wide (excluding the ears) and 15.78 cm high, approximately 9.45° × 15.76° in visual angle. For practice trials, we rendered additional images of one male and one female (Fig. 8c), with either − 24° or 24° eye orientation in the frontal face orientation. The faces for the practice trials were slightly smaller (~ 7.5 cm wide, 11.5 cm high) than those in the main task when presented on screen.

Face images were rendered using Blender 2.79b (The Blender Foundation, Amsterdam, The Netherlands). To create facial images for the main task, we used 3D scan model data of two male and two female faces with an Asian appearance obtained from a 3D scanning company, Ten24 (https://ten24.info/3d-scanning/). The original 3D face models had neutral expressions and varying mouth openings. To ensure uniformly closed lips and a child-friendly appearance, we adjusted each face to a subtle smile by using R3RDS WRAP software. We then fitted 3D eyeball models created in Blender^55^, which simulate human eye anatomy, along with 3D hair and clothing models. In Blender, the orientation of the eyes was controlled by setting fixation targets 45 cm from the face, introducing a 5° lateral offset approximating a typical positive angle kappa^56^. Face images were rendered in Blender 2.70 with a single light source illuminating from above the camera, which was positioned 45 cm from the face and aimed at the midpoint between the eyes.

For the stimulus images of practice trials, we created one female and one male 3D head by morphing three Ten24 scans per sex in R3DS Wrap software. As with for the faces for the main task, we fitted 3D eyeball models and clothing to each face. Eye orientation was controlled similarly to the main task stimuli; however, the fixation target and camera positioned 60 cm from the face with a 3° displacement to mimic the angle kappa for the rendering setting.

#### Procedure

Each participant sat approximately 57 cm from the laptop screen with two response buttons: a blue button with a blue square (left) and a pink button with a pink triangle (right). They first watched an instructional animation (Supplementary Video S1) explaining the task: press the pink button if the eyes looked at the pink triangle, and press the blue button if the eyes looked at the blue square. The animation included Japanese text, and the experimenter provided verbal instructions. Each participant then completed eight practice trials using the most averted eye orientations: 2 gaze orientations (− 24°, 24°) × 2 face identities (one female, one male) × 2 repetitions (random order). Each trial displayed a face centered on the screen with the blue square (left) and pink triangle (right) remaining until a response was provided by the participant. Correct/incorrect feedback was given via cartoon images and sound (Fig. 2b). At the end of the practice trials, the accuracy across the eight practice trials was displayed for 5 s. If the accuracy was < 100%, the participant was give the instructions again and engaged in practice trials until they achieved perfect accuracy.

After achieving 100% accuracy, each participant proceeded to the main task (168 trials): 7 eye orientations (− 24°, − 12°, − 6°, 0°, 6°, 12°, and 24°) × 4 face identities (2 female, 2 male) × 2 image orientations (original, flipped) × 3 face orientations (± 15°, 0°). The flipped images were used to control for facial asymmetries.

The main task was divided into three face-orientation blocks (frontal, leftward, and rightward), with a randomized trial order within each block. The frontal block was the first block for all of the participants, followed by the angled blocks. The order of the leftward and rightward blocks varied across participants and was approximately counterbalanced within each age group. Each block began with a cartoon animation depicting the corresponding face orientation (Supplementary Videos S1, S2 and S3). During the second and third blocks, the experimenter verbally reminded each participant that only the face orientation changed, not the task.

Stimuli appeared as in the practice trials, but no feedback was given. Instead, responses were followed by one of 56 randomly chosen characters and the “correct” sound from the practice session. Every seventh trial, a progress screen appeared (Supplementary Videos S4), allowing breaks before resuming. At the end of each block, a cheering cartoon image marked the completion, and the participant could continue or take a break.

In a subset of the trials, some participants reported difficulty in determining whether the direction was leftward or rightward, perceiving the face as looking directly at them. When this occurred, the experimenter reassured them and encouraged a best guess instead of overthinking.

### Experiment 2

#### Participants

Twenty-four undergraduate students (*M* = 19.67 years, *SD* = 1.09, 16 females), 26 adults aged 20–39 years (*M* = 34.92 years, *SD* = 3.00, 18 females), and 25 adults aged 40–59 years (*M* = 45.80 years, *SD* = 4.21, 22 females) participated in this experiment.

#### Stimuli and procedures

The stimuli and procedures were identical to those in Experiment 1, and the data were analyzed using the same method as that applied in Experiment 1.

### Experiment 3

#### Participants

Twenty-four 4- to 6-year-olds (*M* = 5.62 years, *SD* = 0.87, 12 females), 25 7- to 9-year-olds (*M* = 8.28 years, *SD* = 0.78, 13 females), and 24 10- to 16-year-olds (*M* = 11.80 years, *SD* = 1.71, 13 female) participated in this experiment. An additional seven children were tested but excluded for the following reasons: failure to complete the task (*n* = 1) and failing to reach 75% accuracy for the most averted gaze in any testing block (*n* = 4).

#### Stimuli and procedures

The stimuli and procedures were identical to those used in Experiments 1 and 2, except for the following modifications. We used the Normal images in this experiment (see Fig. 2, bottom row). The frontally oriented faces were identical to those used in Experiment 1. For the angled face images, head orientation was manipulated by rotating the head model within the 3-dimensional environment of Blender 2.79b. The eyeball models remained fixed in their anatomical position within the eye sockets of the head and continuously tracked the same fixation target throughout, ensuring that eye orientation was held constant across head orientations. No post-rendering edits were applied to the Normal images. As a consequence, the appearance of the eye region varied naturally across head orientations due to changes in perspective and partial occlusion, which produced differences in eyelid contour and the relative amount of visible sclera on either side of the iris (see Fig. 2, bottom row).

### Experiment 4

#### Participants

Twenty-five undergraduate students (*M* = 19.76 years, *SD* = 1.16, 17 females) and 26 adults aged 20–59 years (*M* = 42.96 years, *SD* = 4.57 years, 21 females) participated in this experiment. For comparability with Experiment 2, we targeted 24 usable datasets per age group, which exceeds the minimum *n* = 18 indicated by our a priori G*Power analysis (*d* = 0.80, two-tailed; Holm–Bonferroni, *α* = 0.025; power = 0.80).

#### Stimuli and procedures

The stimuli and procedures were identical to those used in Experiment 3.

### Experiment 5

#### Participants

Twenty-four 7- to 9-year-olds participated in the Wollaston condition (*M* = 8.46 years, *SD* = 0.80, 12 females) and 24 participated in the Normal condition (*M* = 8.18 years, *SD* = 0.77 months, 14 females). An additional five children were excluded due to failure to complete the tasks (*n* = 3) or to reach 75% accuracy even in the most averted gaze in any testing block (*n* = 2).

For comparability with Experiments 1 and 3, we targeted 24 usable datasets per condition in the 7- to 9-year-old group, which exceeds the minimum *n* = 18 indicated by an a priori one-sample power analysis (two-tailed; Holm–Bonferroni across two tests, planning at *α* = 0.025; expected effect size *d =* 0.80; power = 0.80).

#### Stimuli and procedures

In Experiment 5, each dimension of the images was reduced to 25% of the size used in Experiments 1–4, resulting in face sizes averaging 2.35 cm in width and 4 cm in height (approximately 2.36° × 4° in the visual angle). Participants were randomly assigned to view either Wollaston or Normal images (Fig. 2). Except for these modifications, all other aspects of the stimuli and procedures remained identical to those in Experiments 1 and 3.

## Statistical analysis

### Dependent variable

The primary dependent variable was the PSE half-difference, defined as the difference between the PSEs obtained from the two opposite head orientation conditions divided by two. Positive values indicate that perceived gaze direction was biased toward the head orientation, whereas negative values indicate a bias opposite to the head orientation. PSEs were estimated for each participant and for each head orientation by fitting a psychometric function to the proportion of “rightward” responses across eye orientation.

### Independent variables

Across Experiments 1–4, the main independent variable for group-level comparisons was age group. In Experiment 5, the independent variables were image size and image condition (Wollaston vs. Normal). Head orientation and eye orientation varied within participants and were used to derive PSEs but were not subjected to additional factorial analyses.

### Analysis approach

PSE half-differences were compared across age groups using Welch’s ANOVA, chosen because preliminary tests indicated variance heterogeneity across age groups. One-sample t-tests were used to evaluate whether PSE half-differences significantly differed from zero (chance). To assess age-related trends, Spearman’s rank-order correlation was used because PSE estimates showed variance heterogeneity between the age groups. Multiple comparisons were corrected using the Holm–Bonferroni method.

### Sample size estimation

#### Experiment 1

Following the report of a Wollaston effect *d*-value of 1.95 by Otsuka et al.^18^ and accounting for our fewer trials with fewer stimulus eye orientations and the greater variability expected in children, we adopted a conservative expected effect size of *d =* 0.80 for the a priori power analysis. We used G*Power (one-sample *t*-test, “Means: Difference from constant,” two-tailed) for the a priori power analysis. For planning, we applied the most stringent adjusted threshold (*α* = 0.05/3 = 0.0167; power = 0.80) using the Holm–Bonferroni correction across three simultaneous tests within an experiment. This analysis indicated a minimum required sample size of *n* = 20 participants per age group. To ensure adequate precision and to accommodate exclusions, we planned to stop collecting data once 24 usable datasets per group had been obtained. Due to scheduling constraints, we ultimately obtained one additional usable dataset in each of the two younger age groups in this experiment.

#### Experiment 2

For comparability with Experiment 1, we targeted 24 usable datasets per group, which exceeds the minimum *n* = 20 indicated by our a priori G*Power analysis (*d* = 0.80; two-tailed; Holm–Bonferroni, *α* = 0.0167; power = 0.80). Due to scheduling constraints, we ultimately obtained two additional datasets for adults aged 20–39 years and one additional dataset for adults aged 40–59 years in this experiment.

#### Experiment 3

Following the report by Otsuka et al.^18^ of a repulsive effect *d =* 1.59 and for comparability with Experiment 1, we targeted 24 usable datasets per group, exceeding the minimum *n* = 20 from the same a priori G*Power analysis (*d =* 0.80; *α* = 0.0167; power = 0.80). Due to scheduling constraints, we ultimately obtained one additional dataset for 7- to 9-year-olds in this experiment.

#### Experiment 4

For comparability with Experiment 2, we targeted 24 usable datasets per group, which exceeds the minimum *n* = 18 indicated by our a priori G*Power analysis (*d* = 0.80, two-tailed; Holm–Bonferroni, *α* = 0.025; power = 0.80).

#### Experiment 5

For comparability with Experiments 1 and 3, we targeted 24 usable datasets per condition in the 7- to 9-year-old group, which exceeds the minimum *n* = 18 indicated by an a priori one-sample power analysis (two-tailed; Holm–Bonferroni across two tests, planning at *α* = 0.025; expected effect size *d* = 0.80; power = 0.80).

## Supplementary Information

Below is the link to the electronic supplementary material.

Supplementary Material 1

Supplementary Material 2

Supplementary Material 3

Supplementary Material 4

Supplementary Material 5

Supplementary Material 6

Supplementary Material 7

## Data Availability

Due to licensing restrictions, the stimulus images cannot be publicly shared. However, they may be made available upon request for academic, non-commercial inspection purposes only, and under a non-redistribution agreement. The anonymized dataset used in this study is available at the Open Science Framework (https://osf.io/acdjk/?view_only=a190bcdd59bf41799166bdabf8940c65).
