# Supplementary figures and images for: Developmental trajectories of head and eye cue integration in gaze perception
Source: Sci Rep. 2026 Jan 6;16:4465. doi: 10.1038/s41598-025-34625-9 (PMC12865040; doi:10.1038/s41598-025-34625-9)

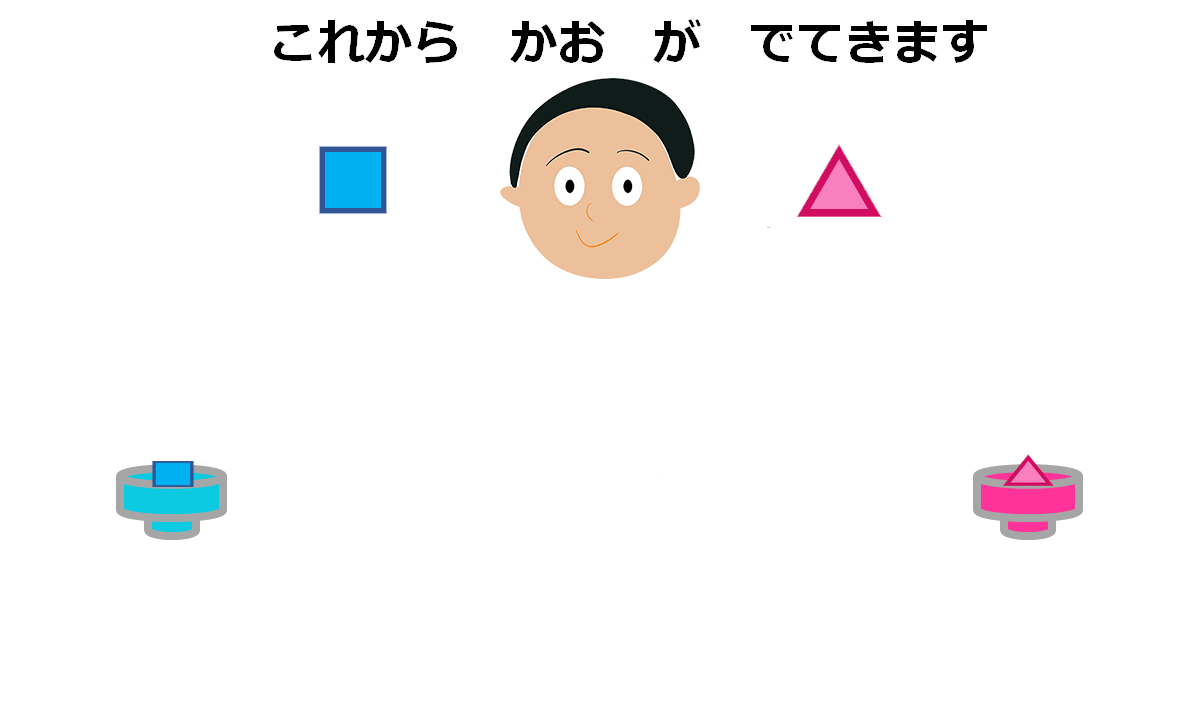

Supplement: Supplementary file 1 — Supplementary Material 1 [file 41598_2025_34625_MOESM1_ESM.gif]

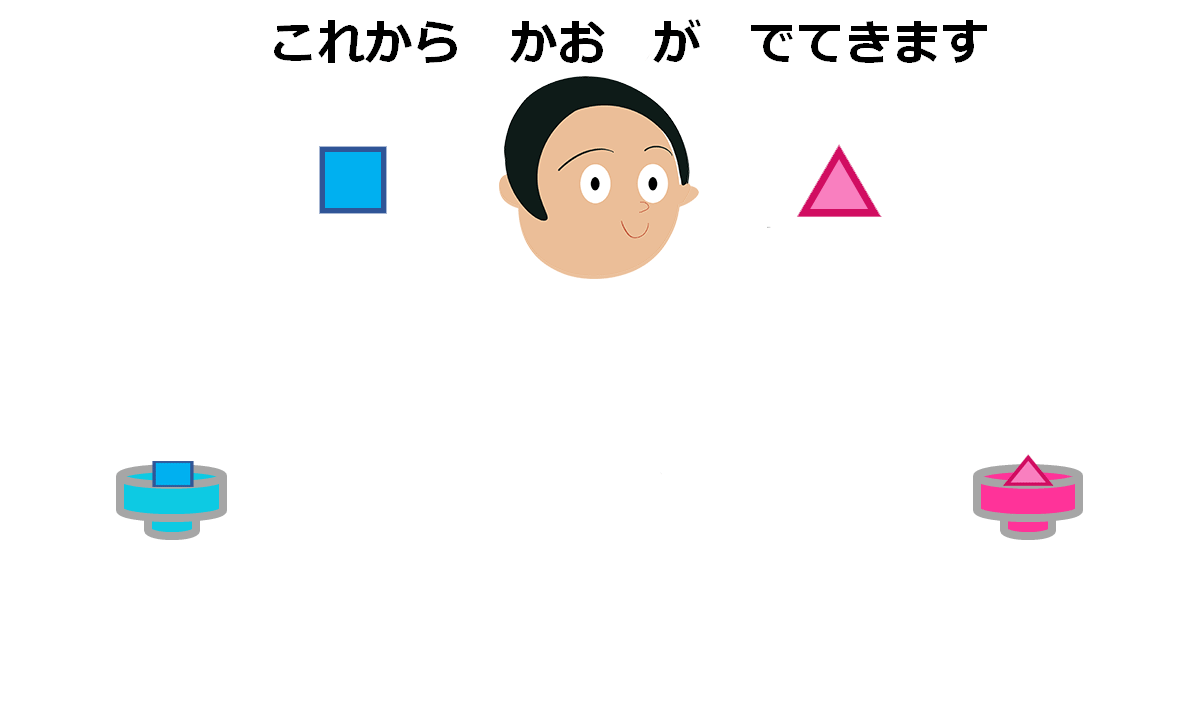

Supplement: Supplementary file 2 — Supplementary Material 2 [file 41598_2025_34625_MOESM2_ESM.gif]

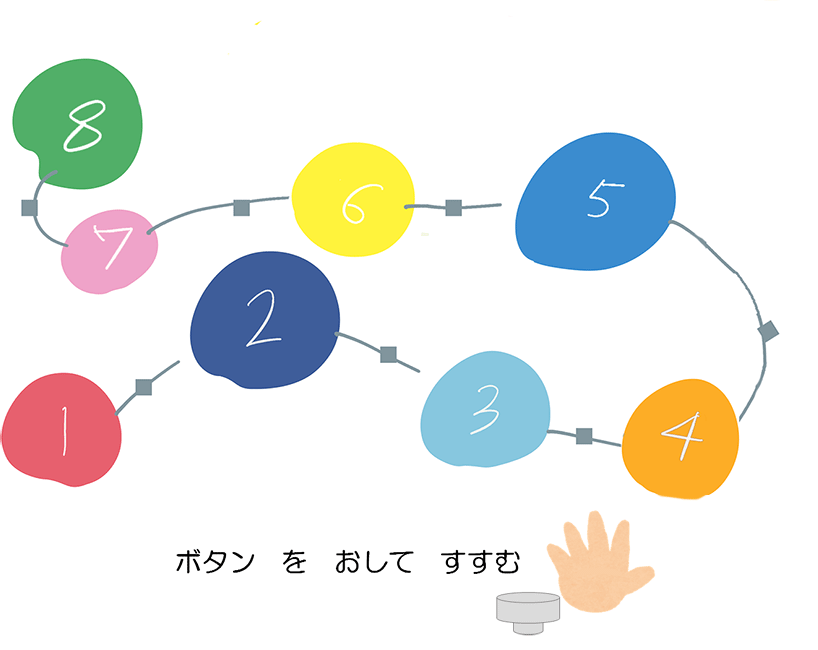

Supplement: Supplementary file 3 — Supplementary Material 3 [file 41598_2025_34625_MOESM3_ESM.gif]

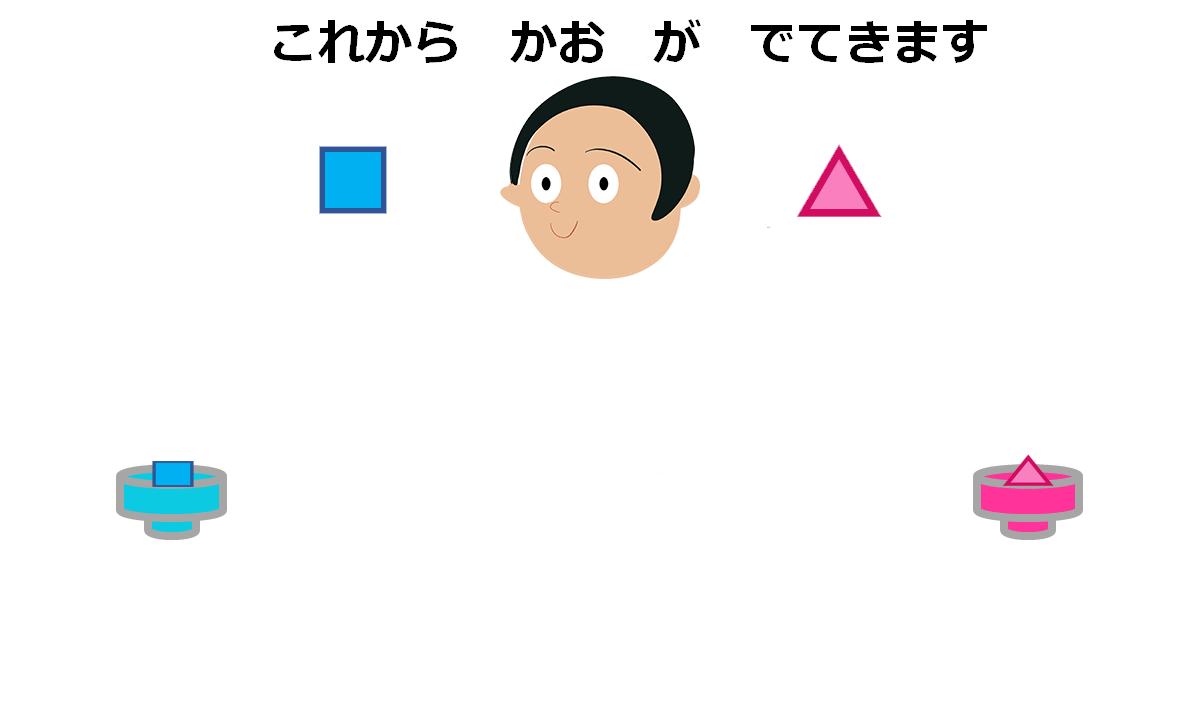

Supplement: Supplementary file 4 — Supplementary Material 4 [file 41598_2025_34625_MOESM4_ESM.gif]
