# Supplementary material for: Developmental trajectories of head and eye cue integration in gaze perception
Source: Sci Rep. 2026 Jan 6;16:4465. doi: 10.1038/s41598-025-34625-9 (PMC12865040; doi:10.1038/s41598-025-34625-9)
Supplement: Supplementary file 5 — Supplementary Material 5 [file 41598_2025_34625_MOESM5_ESM.docx]

**Supplementary Note 1**

During the testing for the current study, some participants reported difficulty distinguishing left from right, stating that the face appeared to be looking directly at them. Other participants appeared to exhibit similar difficulties behaviorally, despite not providing explicit reports. Based on these observations, we analyzed reaction times as a function of stimulus eye and head orientation, measured from stimulus onset. We hypothesized that perceived direct gaze would delay categorization.

For each participant, we calculated the median reaction time for each eye and head orientation, then averaged these across groups. Supplementary Figure S1a shows Gaussian fits to mean reaction times. Reaction time peaks were most pronounced for the frontal head orientation, with peaks near 0° across ages. In younger groups, the peaks for angled head orientation conditions shifted opposite to head orientation.

We utilized peak reaction time shifts as an additional measure of perceived gaze shift, complementing the PSE-half difference. Because individual fits sometimes failed, we applied Gaussian fitting to group-level data. To test whether the average Peak-half difference significantly deviated from 0, we employed bootstrapping with 2000 iterations. Each iteration involved resampling participants, recalculating mean reaction times, refitting the Gaussian curve, and computing the Peak-half difference.

**Reaction time results from Experiment 1 (Wollaston images with children)**

Supplementary Figure S1b shows the median bootstrapped Peak-half difference in RT with 95% confidence intervals for each age group. As with the PSE-half difference, a positive Peak-half difference in RT indicates an attractive influence of head orientation, while a negative value indicates a repulsive influence. Thus, if the Wollaston illusion occurs, the Peak-half difference in RT is expected to be positive. We computed two-tailed *p*-values for bootstrapped Peak-half differences in RT against 0 for each age group, applying Holm-Bonferroni correction. A significant positive shift was found in 4- to 6-year-olds (*p* = .046) and 7- to 9-year-olds (*p* = .003), but not in 10- to 16-year-olds (*p* = .324).

**Supplementary Figure S1.** *Reaction times for each age group in Experiment 1.* (a) Mean reaction times as a function of stimulus eye orientation and head orientation, with Gaussian fits. Vertical dashed lines mark reaction time peaks. Error bars represent ±1 SEM. (b) Bootstrapped median Peak-half RT difference. Error bars represent 95% CI. (c) Bootstrapped median sigma parameter for frontal head orientation. Error bars represent 95% CI.


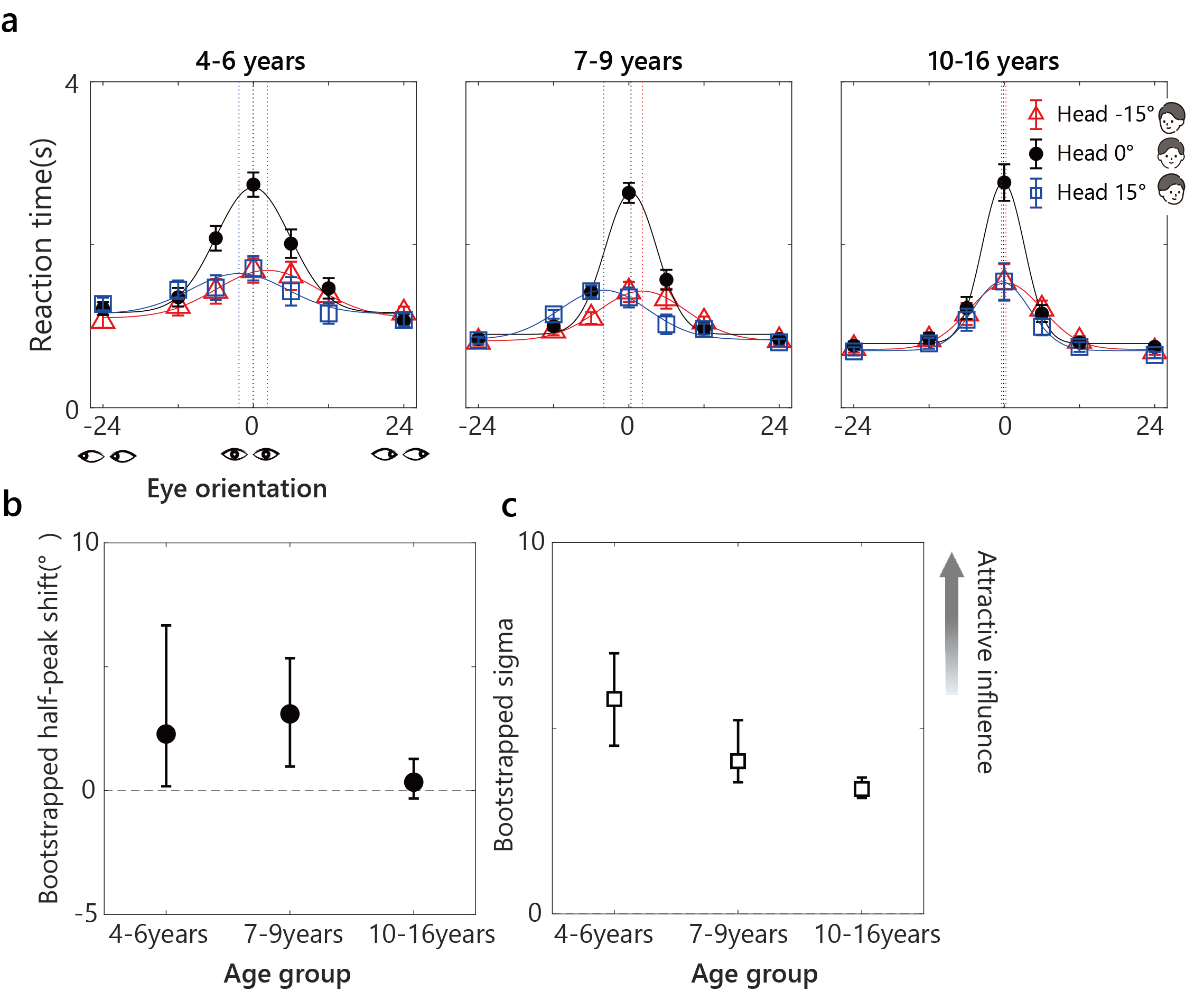


Past studies suggest the perceived range of "direct" gaze narrows with age [29-30]. Similarly, Supplementary Figure S1a shows that the RT Gaussian curve for frontal head orientation is widest in 4- to 6-year-olds and narrows with age. To test this trend, we analyzed the sigma parameter, reflecting curve spread (Supplementary Figure S1c). Bootstrapped comparisons showed sigma was significantly smaller in 10- to 16-year-olds than in younger groups (vs. 4–6 years: *p* < .01; vs. 7–9 years: *p* = .012) but did not differ between 4- to 6 and 7- to 9-year-olds (*p* = .072). These findings align with previous research, suggesting that younger children perceive a broader range of gaze directions as direct.

**Reaction time analysis for Experiment 2 (Wollaston images with adults)**

The Peak-half difference in RT (Supplementary Figure S2b) was significantly shifted from 0 in adults aged 20–59 years (20-39 years: *p* < .001; 40-59 years: *p* < .001) but not in students (**≈**20 years: *p* = .874). Because the student group showed a significant PSE-half difference in rightward responses, the lack of a significant Peak-half difference suggests a weaker Wollaston effect in this group. Supplementary Figure S2c shows the median bootstrapped sigma for each group. No significant differences were found in sigma for frontal head orientation among adults (students **≈**20 years vs. 20-39 years: *p* = 1.227; students **≈**20 years vs. 40-59 years: *p* = .834; 20-39 years vs. 40-59 years: *p* = .84), indicating that the range of gaze perceived as “direct” remains stable in adulthood.

**Supplementary Figure S2**. *Reaction times for each age group in Experiment 2.* (a) Mean reaction times with Gaussian fits. Error bars represent ±1 SEM. (b) Bootstrapped median Peak-half RT difference. Error bars represent 95% CI. (c) Bootstrapped median sigma parameter for frontal head orientation. Error bars represent 95% CI.


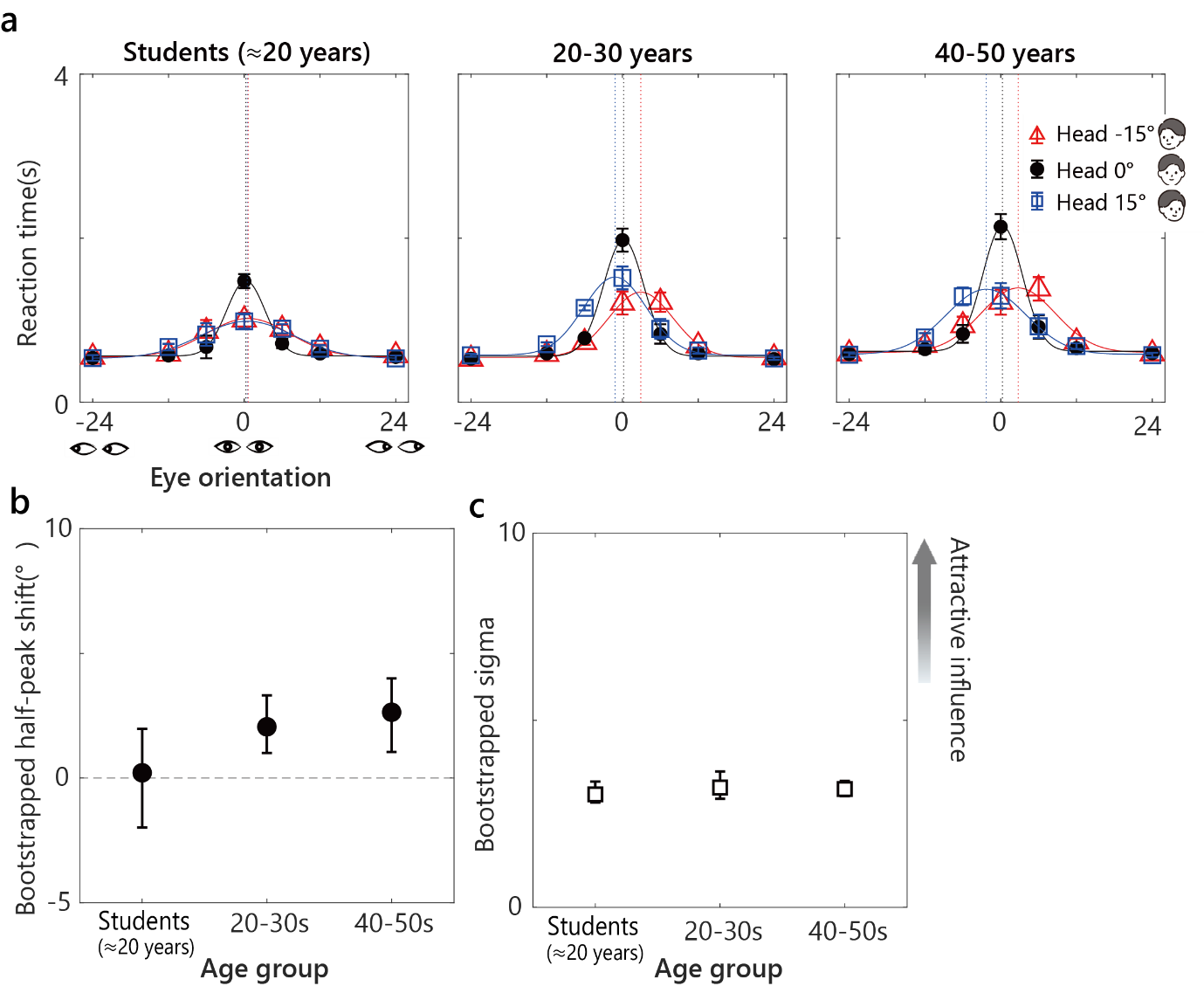


**Reaction time analysis for Experiment 3 (Normal images with children)**

Peak-half differences in RT were significantly negative across all ages (*p*s < .001; Supplementary Figure S3b), indicating a strong repulsive effect of head orientation, consistent with the PSE-half difference reported in the main text. As in Experiment 1, the RT Gaussian curve for frontal head orientation was widest in 4- to 6-year-olds and narrowed with age (Supplementary Figure S3a). Bootstrapped sigma comparisons (Supplementary Figure S3c) showed a significant difference between 4- to 6-year-olds and 10- to 16-year-olds (*p* = .012) but not between other groups (4–6 years vs. 7–9 years: *p* = .15; 7–9 years vs. 10–16 years: *p* = .22). These findings support previous research suggesting younger children perceive a broader range of gaze directions as direct [29-30].

**Supplementary Figure S3.** *Reaction times for each age group in Experiment 3.* (a) Mean reaction times with Gaussian fits. Error bars represent ±1 SEM. (b) Bootstrapped median Peak-half RT difference. Error bars represent 95% CI. (c) Bootstrapped median sigma parameter for frontal head orientation. Error bars represent 95% CI.

*
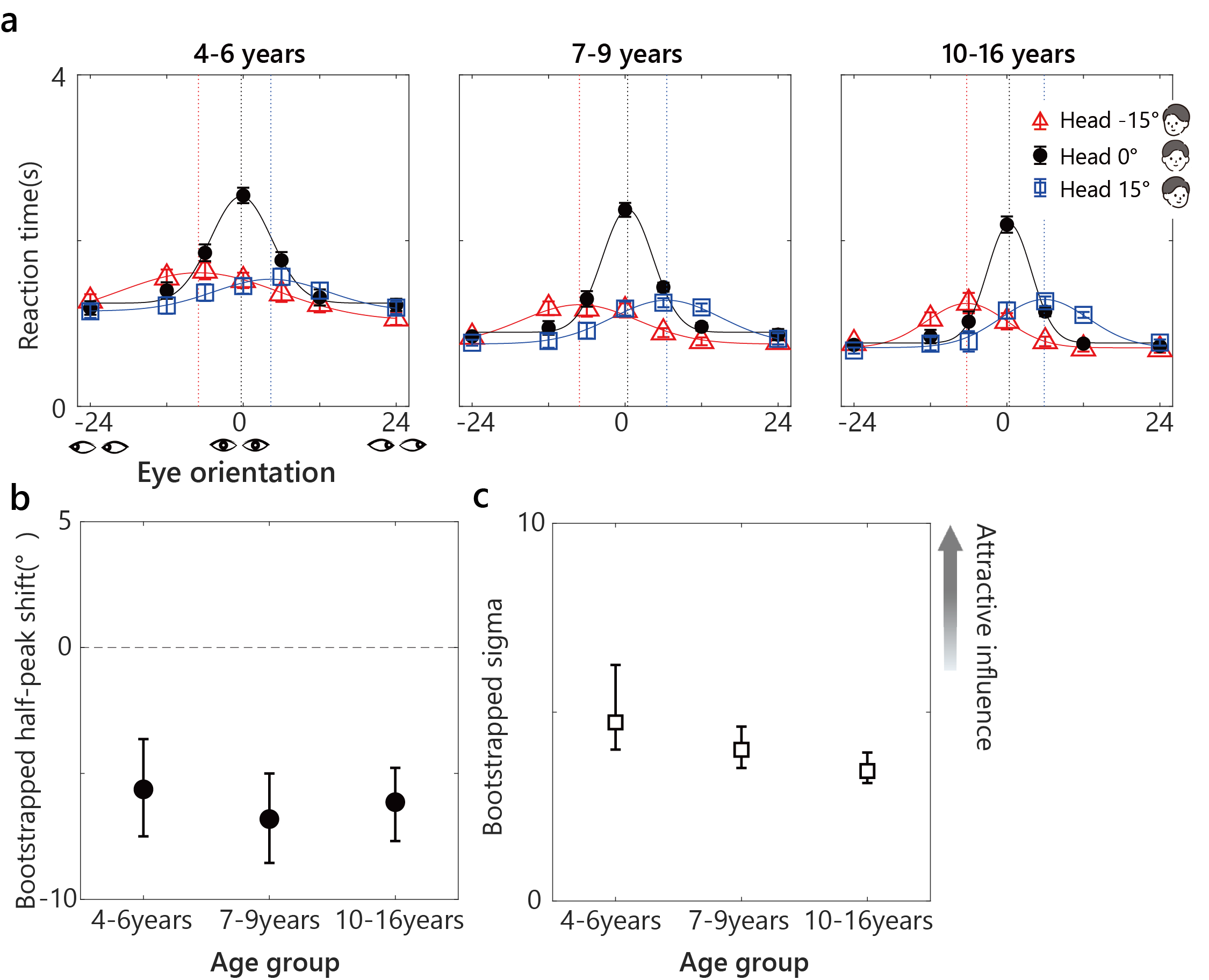
*

**Reaction time analysis for Experiment 4 (Normal images with adults)**

The Peak-half difference in RT was significantly negative in both groups (students ≈20 years: *p* = .019; 20-59 years: *p* < .001, Supplementary Figure S4b), indicating a repulsive gaze shift away from head orientation, consistent with the PSE-half difference reported in the main text. No significant sigma difference was found between groups for frontal head orientation (*p* = .821, Supplementary Figure S4c).

**Supplementary Figure S4.** *Reaction times for each age group in Experiment 4.* (a) Mean reaction times with Gaussian fits. Error bars represent ±1 SEM. (b) Bootstrapped median Peak-half RT difference. Error bars represent 95% CI. (c) Bootstrapped median sigma parameter for frontal head orientation. Error bars represent 95% CI.


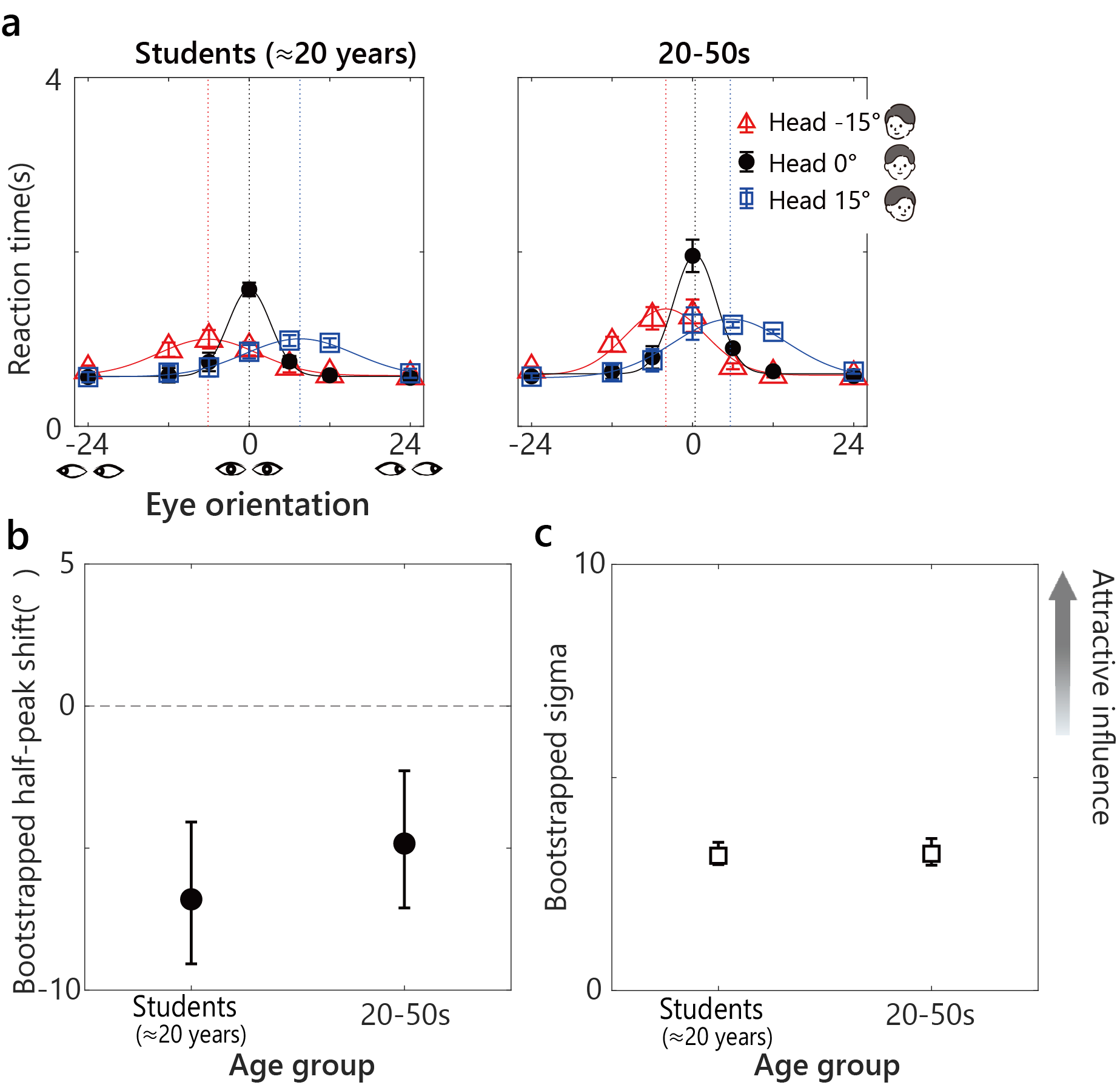


**Reaction time analysis for Experiment 5 (small images with children)**

The Peak-half difference in RT was significantly positive in the Wollaston condition and negative in the Normal condition (*p*s < .001, Supplementary Figure S5), consistent with the PSE-half difference reported in the main text. This suggests gaze perception was attracted to head orientation in the Wollaston condition but repelled in the Normal condition. Supplementary Figure S5b shows the median bootstrapped sigma for each group.

**Supplementary Figure S5.** *Reaction times for each image condition in Experiment 5.* (a) Mean reaction times with Gaussian fits. Error bars represent ±1 SEM. (b) Bootstrapped median Peak-half RT difference. Error bars represent 95% CI. (c) Bootstrapped median sigma parameter for frontal head orientation. Error bars represent 95% CI.

**
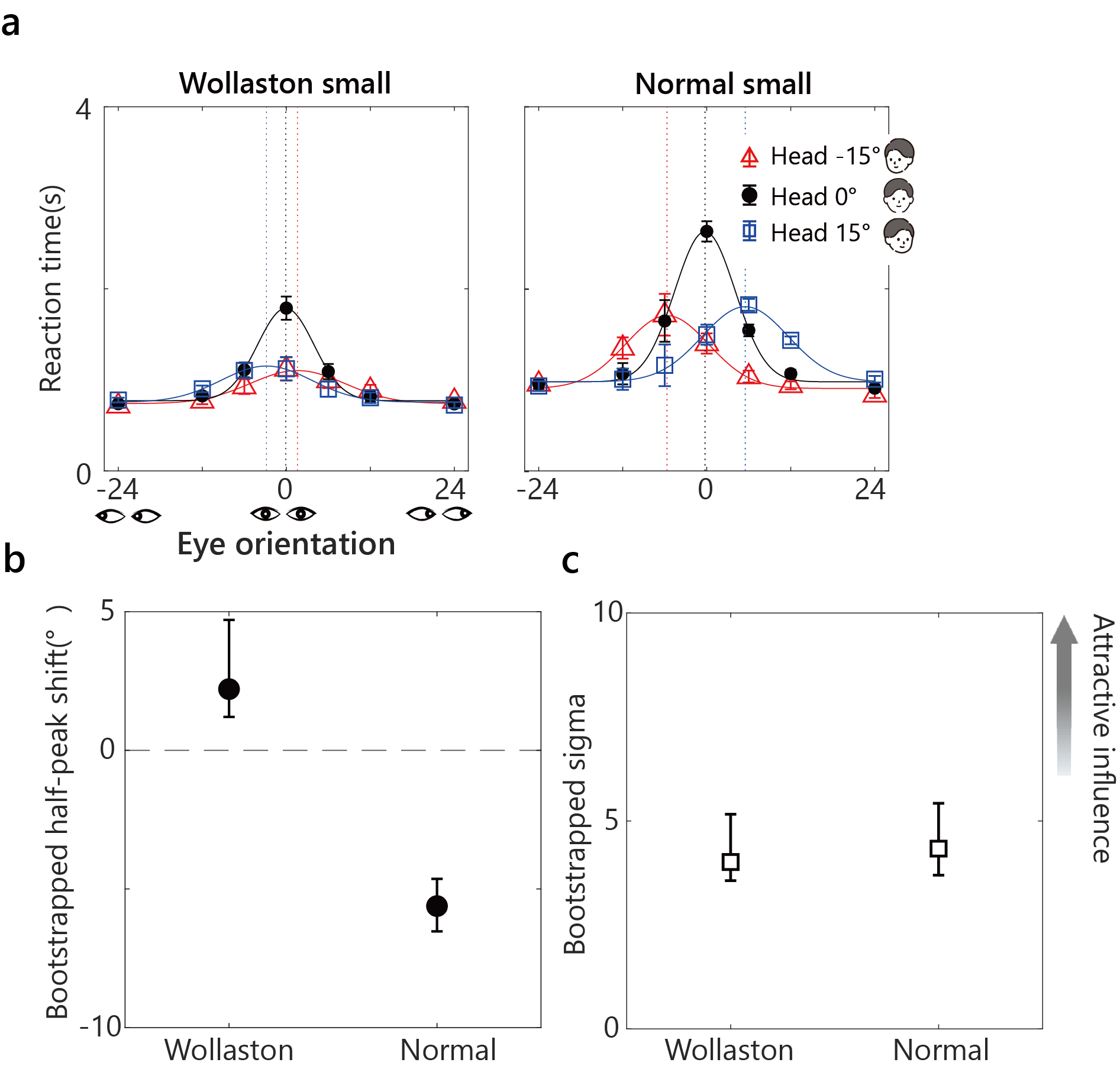
**
