# Supplementary material for: Developmental trajectories of head and eye cue integration in gaze perception
Source: Sci Rep. 2026 Jan 6;16:4465. doi: 10.1038/s41598-025-34625-9 (PMC12865040; doi:10.1038/s41598-025-34625-9)
Supplement: Supplementary file 6 — Supplementary Material 6 [file 41598_2025_34625_MOESM6_ESM.docx]

**Supplementary Videos**

**Supplementary Video S1.** Instructional animation for the gaze judgment task. This GIF shows the animated instructions presented to participants before the practice trials and before the frontal face orientation block of the main task.

**Supplementary Video S2.** Animation indicating the leftward head orientation block. This GIF illustrates the cartoon animation shown at the beginning of the block with leftward head orientation of the main task.

**Supplementary Video S3.** Animation indicating the rightward head orientation block. This GIF illustrates the cartoon animation shown at the beginning of the block with rightward head orientation of the main task.

**Supplementary Video S4.** Progress screen animation. This GIF shows the progress screen animation presented every seventh trial during the main task.
