# Supplementary material for: Developmental trajectories of head and eye cue integration in gaze perception
Source: Sci Rep. 2026 Jan 6;16:4465. doi: 10.1038/s41598-025-34625-9 (PMC12865040; doi:10.1038/s41598-025-34625-9)
Supplement: Supplementary file 7 — Supplementary Material 7 [file 41598_2025_34625_MOESM7_ESM.docx]

**Supplementary Note 2**

To examine the possibility that adolescents have higher precision in processing eye region information, we compared the slope of the psychometric function for rightward responses across the age groups of children and adults, analyzing Wollaston (Experiments 1 and 2) and Normal images (Experiments 3 and 4) separately. Higher precision in processing eye region information is expected to manifest as a steeper slope in the psychometric function, reflecting more precise discrimination of gaze deviations. For each image type, we conducted a two-way ANOVA on the slope, with head orientation (frontal vs. angled) as a repeated measure and age group as a between-subject factor. For Wollaston images, the ANOVA revealed a significant main effect of head orientation, *F* (2, 265.946) = 20.86, *p* < .001, *η*²_p_ = 0.127, with steeper slopes for frontal compared to angled faces. There was also a significant main effect of age, *F* (5, 143) = 12.92, *p* < .001, *η*²_p_ = 0.31. Post hoc comparisons showed shallower slopes in the two younger child groups compared to adults (4–6 years: *p* < .001; 7–9 years: *p* = .002), whereas the 10- to 16-year-olds showed slopes comparable to adults (*p* > .1). For Normal images, the ANOVA similarly revealed a significant main effect of head orientation, *F* (2, 238) = 10.64, *p* < .01, *η*²_p_ = 0.082, and a significant main effect of age, *F* (4, 119) = 14.126, *p* < .01, *η*²_p_ = 0.322. The post hoc pattern was the same as in the Wollaston condition.
